# Genome-wide investigation of prosody perception: Shared genetic influences between speech rhythm, musical rhythm, and reading traits

**DOI:** 10.1016/j.xhgg.2026.100581

**Published:** 2026-02-18

**Authors:** Alyssa C. Scartozzi, Youjia Wang, Peyton L. Coleman, Ximena León Du’Mottuchi, Tara L. Henechowicz, Daniel E. Gustavson, Lauren E. Petty, Heather M. Highland, Nicole Creanza, Cyrille L. Magne, Rosa S. Gísladóttir, Nancy J. Cox, Jennifer E. Below, Srishti Nayak, Reyna L. Gordon

**Affiliations:** 1 Vanderbilt Genetics Institute, Vanderbilt University Medical Center, Nashville, TN 37203, USA; 2 Department of Otolaryngology - Head & Neck Surgery, Vanderbilt University Medical Center, Nashville, TN 37232, USA; 3 Medical College of Wisconsin, Department of Internal Medicine and Pediatrics, Milwaukee, WI 53226, USA; 4 Center for Digital Genomic Medicine, Vanderbilt University Medical Center, Nashville, TN 27203, USA; 5 Department of Biological Sciences, Vanderbilt University, Nashville, TN 37203, USA; 6 Institute for Behavioral Genetics, University of Colorado Boulder, Boulder, CO 80303, USA; 7 Department of Epidemiology, UTHealth Houston, Houston, TX 77030, USA; 8 Department of Psychology, Middle Tennessee State University, Murfreesboro, TN 37132, USA; 9 Department of Icelandic and Comparative Cultural Studies, University of Iceland, Saemundargata 2, 102 Reykjavik, Iceland

**Keywords:** genome-wide association study, complex genetics, prosody, speech rhythm perception, language

## Abstract

Prosody perception is an often overlooked aspect of human language despite its importance in facilitating spoken language comprehension. Sensitivity to prosodic cues varies between individuals, and prosody perception skills are shown to be associated with various language- and reading-related outcomes. Despite the importance of prosody perception in human communication, its underlying biology is poorly understood. This study investigates the genetic architecture of prosody (speech rhythm) perception and explores its evolutionary roots. We conducted a GWAS of prosody (*n* = 1,501) as measured by scores on the Test of Prosody via Syllable Emphasis (“TOPsy”). GWAS results yielded 14 suggestive significant signals (*p* < 5.00 × 10^−6^). Gene set enrichment analysis identified shared genetic architecture between human prosody perception and key vocal learning brain regions in songbirds, suggesting that human prosody perception may have evolutionary convergence in communication mechanisms in animal vocal learning. Additionally, cross-trait polygenic score analyses suggest shared genetic influences between prosody perception and both word reading and musical beat synchronization, emphasizing how genetics influence prosody perception and its associations with communication-, education-, and music-related traits. These initial efforts could inform advances in communication sciences and disorders as well as educational contexts.

## Teaser

Speech rhythm (prosody) perception is an important aspect of language and reading development. We found that genetic predispositions for musical rhythm and reading were associated with speech rhythm perception skills. The genetics of human prosody perception were enriched in key songbird vocal learning brain regions, shedding light on evolutionary origins.

## Introduction

Our capacity to communicate using spoken language, which we generally learn from a young age, is one of the most remarkable features of the human mind. While most research on language traits has focused on neuroscience and behavioral methods, there is emerging interest in the potential genetic influences on human language development and function. Twin- and family-based studies show that there are moderate to strong genetic influences on several speech and language traits, with heritability estimates ranging from 0.46 to 0.97 (see Nayak et al.^1^ for a brief review). Although genetic investigations of language and disorders have primarily focused on family and linkage-type analyses, recent genome-wide association studies (GWASs) show that many speech, language, and reading-related traits and disorders (i.e., reading-related traits,^2^ speech acoustics,^3^ dyslexia,^4^ and stuttering^5^^,^^6^^,^^7^) are moderately influenced by common genetic variation. For example, up to 13% of the variation in nonword repetition (a task characterized by listening and repeating back nonsense/meaningless words) is explained by differences in common genetic variants.^2^ These studies suggest that language-related traits are polygenic, complex, and moderately distinct from other cognitive and educational traits,^8^ with some degree of shared genetic architecture with other brain, health, and behavioral phenotypes.^9^ Moreover, genetic variation plays a role in the development, structure, and function of the brain’s language network.^2^^,^^9^^,^^10^

An often overlooked aspect of language is prosody, defined as the intonational and durational patterns within spoken language. Speech rhythm perception is a key aspect of prosody that involves the recognition of stress patterns in language. For example, the meaning of the sentence “John bought the car” changes depending on where the stress is placed (e.g., “*John* bought the car” [not Fred] or “John bought the *car*” [not motorcycle]). This intricate skill allows us to detect variations in emphasis within words, sentences, and larger spoken language structures. Sensitivity to spoken stress patterns emerges from infancy^11^ and becomes particularly significant during mid-childhood and elementary education. During this period, children draw on speech rhythm cues to enhance crucial linguistic processes encompassing lexical retrieval,^12^ morpho-syntactic parsing,^13^ and understanding the intended meaning of a sentence beyond the literal words.^14^ Additionally, speech rhythm perception skills are highly related to success in learning to read^15^^,^^16^^,^^17^^,^^18^; and its importance persists into adult literacy outcomes^19^ and silent reading representations, where readers project prosodic changes onto text read silently (“Implicit Prosody Hypothesis”).^20^^,^^21^ Together, these studies highlight the importance of prosody in reading and language outcomes across all ages.

While traditional measures of phonological skill do not typically include an isolated assessment of prosodic skill, we can gain insight into the genetic underpinnings of prosody, which is a facet of linguistic phonology (i.e., rules governing how units of sound in speech go together in a language),^22^ from studies of phonology. To our knowledge, there are no heritability studies focused purely on prosody perception. However, given the shared reliance of both prosody and phonological processing on underlying mechanisms such as auditory discrimination and working memory, heritability estimates for phonological awareness, which range from 0.46 to 0.64,^23^^,^^24^ suggest that a significant heritable component likely contributes to prosodic skills. We use these estimates as the closest available proxy for the heritability of general speech rhythm perception. Genomic studies of prosodic skills, including speech rhythm perception, may link to reading and other speech-language traits that have been more robustly genetically characterized. Further, uncovering a shared genetic architecture of prosody, speech, language, reading, and/or rhythm could inform education solutions for improved reading and literacy outcomes.

The emerging body of work on the genetic underpinnings of prosody may also have implications for the evolutionary history of language traits. It has been theorized that early communication through prosody-rich protolanguage sounds facilitated the survival of groups, and ultimately led to the emergence of even more complex organized patterns (i.e., syntax^25^) in humans. This gradual, pre-adaptive process could have leveraged existing genetic architecture, conserving genetic variation associated with auditory-vocal processing (i.e., the ability to make or perceive these sounds) due to survival benefits (see Kotz et al.^26^ for a review of evolution of rhythm processing). Vocal communication in species capable of vocal learning (i.e., species that can modify their own vocalizations as a result of experience with those of other individuals^27^^,^^28^^,^^29^) displays a number of features similar to human prosody. The connection between songbirds and humans in particular is noteworthy in that both have the capacity to learn and communicate using complex patterns of sounds (“Revised Vocal Learning Hypothesis”^30^^,^^31^). These behavioral similarities are further complemented through similar neurobiological structure and function supporting songbird vocal learning and human communication abilities, including at the genetic level.^32^^,^^33^^,^^34^ Specifically, the comparative analysis conducted by Mol et al.^35^ highlights significant similarities between the suprasegmental organization of human prosody and songbird song. They demonstrate that they both share a hierarchical structure, where fundamental acoustic units are organized into larger, phrase-like groups (e.g., syllables are grouped into motifs, and words are organized into phonological phrases). Importantly, the temporal and melodic characteristics of these structures are influenced by context-dependent acoustic variations that convey essential social and emotional information. Furthermore, both human infants and juvenile songbirds depend significantly on these prosody-like acoustic cues to segment and learn complex vocal signals during their respective developmental stages.

In addition to human language and communication analogs to songbird vocal learning, there is also evidence that vocal learning and human rhythm processing (e.g., beat synchronization) share some degree of common genetic substrates. Given that prosody perception as defined here (in terms of word-level stress patterns) is a fundamentally rhythmic element of spoken language processing, the current work extends these comparative analyses between humans and songbirds (the largest evolutionary radiation of vocal learners) to the domain of prosody perception. Building upon prior evidence, particularly considering evolutionary adaptation and conservation of shared genetic signals, we hypothesize some overlap in the genetic substrates of prosody and songbird vocal learning.

Indeed, underdeveloped sensitivity to speech rhythm perception may be a key component of reading disorders, given that on average both children and adults with dyslexia perform less well on tasks that involve manipulating stress patterns of words than those without dyslexia.^36^^,^^37^^,^^38^ Impaired rhythm processing, across both speech rhythm and musical rhythm domains, potentially involves less efficient neural subprocesses such as precise auditory timing, synchronization of neural oscillatory activity, and sensorimotor coupling. These subprocesses are hypothesized to play a downstream role in reading and other language-related impairments.^39^^,^^40^ Behaviorally, individual differences in speech rhythm perception tasks have been explained in part by variance in musical rhythm perception abilities^41^^,^^42^^,^^43^ pointing to domain-general rhythm processes. Interestingly, recent theories further posit widespread shared genetic influences between musical rhythm and human speech-language traits including prosody perception.^1^ A recently published large genetic study of musical rhythm (specifically, the self-reported ability to clap in time with a musical beat, i.e., “beat synchronization”) revealed 69 genome-wide significant loci, and downstream biological discovery of musical rhythm abilities in humans.^44^ Musical rhythm traits are directly biologically relevant to language abilities as evidenced by behavioral^1^^,^^45^ and genetic findings.^7^^,^^9^ Further, musical rhythm abilities are related specifically to prosody perception.^1^^,^^41^^,^^42^^,^^43^ This recent GWAS of musical rhythm abilities motivates further genetic investigations of prosody in the current work.

Despite its importance for reading and language development and disorders and educational outcomes, the biology of prosody is sparsely understood. In order to gain insights into its biology, and test theories about biological and evolutionary connections between musical rhythm, speech rhythm, and language/reading abilities, we first need to systematically characterize the genetics of prosody perception. Characterizing the genetics of prosody, and consequently understanding the biological associations between prosody, reading, and other rhythm and language-related traits may offer valuable insights into how prosody influences reading outcomes, as well as other speech-language development outcomes. The current study investigates the genetic architecture of speech rhythm perception using population-based genome-wide approaches. We performed a GWAS of a validated speech rhythm perception phenotype in *n* = 1,501 individuals of European genetic ancestry to identify genomic loci associated with prosody perception scores. To begin to explore the potentially shared evolutionary history between songbird and human communication, we performed gene set enrichment analysis using songbird vocal learning gene sets. Last, to evaluate the genetic relationships between prosody and other phenotypically relevant traits, we estimated if polygenic scores (PGSs) for (1) word reading, (2) beat synchronization, and (3) voice pitch variability in reading (which correlates phenotypically with verbal fluency and reading measures),^3^ predict prosody perception scores in an independent sample. Together, this research is an important step toward understanding shared etiological relationships between prosody and other speech, language, and literacy outcomes.

## Subjects and Methods

### Phenotyping

Test for Prosody via Syllable Emphasis (“TOPsy”) is a 28-item word-level stress identification task that requires participants to identify which syllable carries the primary emphasis or stress in a series of multi-syllabic spoken words.^41^ TOPsy is a highly reliable and validated task that was specifically designed to improve phenotyping in the context of large-scale genomics research. TOPsy has several features that address common trade-offs between characterizing phenotypes richly, and achieving large sample sizes. For instance, TOPsy allows for remote testing and automatic scoring (not possible for existing prosody perception tests), while improving reliability compared with other existing prosody tests (Cronbach’s α = 0.92). Further, TOPsy counterbalances syllable length and syllable stress position across items tested, a feature not previously incorporated into other prosody perception tests.^19^^,^^46^ The 28 test items were finalized through exploratory factor analysis, and the test takes 10 min to complete remotely, making it ideal for human genomics research. Development and validation of TOPsy were previously reported elsewhere (see Nayak et al.^41^).

### Study design

The study population was a subset of those who participated in the *Vanderbilt Online Musicality Study*,^47^ where participants completed various internet-based tasks, including TOPsy, and submitted genetic samples. A total of 1,698 participants (*n* = 1,501 with European genetic ancestry) completed the TOPsy task. Due to substantial skew in the data, data from the TOPsy task were inverse-rank transformed. Specifically, a linear model was fit adjusting for age, sex, and first five ancestry principal components (PCs); and residuals of this model were inverse-rank transformed, and used as the new speech rhythm perception phenotype. All analyses were performed focusing on those with European genetic ancestry, and using transformed speech rhythm perception (TOPsy) scores, unless otherwise specified (i.e., in PGS analyses). All participants provided informed consent prior to participation, and study procedures were approved by Vanderbilt's Institutional Review Board.

### Genotyping, quality control procedures, and imputation

Participants were genotyped using the Illumina Expanded Multi-Ethnic Genotyping Array (MEGA^EX^) at VANTAGE, Vanderbilt University Medical Center’s core facility. Genetic data quality was assessed and missing variants were imputed as part of the Vanderbilt Online Musicality Study (see supplement in Gustavson et al.^47^ for more details).

Briefly, sample and variant filtering was performed in PLINK v.1.90^48^ using the following parameters: minor allele frequency <0.01, variant missingness > 0.10, and sample missingness > 0.15. Samples that failed the heterozygosity (F > 0.2 or < -0.2) and sex checks (mismatch in imputed and expected biological sex) were removed. PC analysis was performed on the maximum unrelated set using PC-Air to determine genetic ancestry.^49^ Kinship was then estimated with PC-Relate.^50^ After, the data were filtered again to remove samples with missingness > 0.05 and Hardy-Weinberg equilibrium *p* < 1 × 10^−8^. Data were then phased using Eagle v.2.4^51^ and then imputed to TOPMed Imputation Server^52^ using Minimac4.^53^ Post-imputation quality control filtering consisted of removing variants with a minor allele frequency < 0.01 and imputation quality R^2^ < 0.70 using BCFtools.^54^ Using this cleaned dataset from the Vanderbilt Online Musicality Study,^47^ we identified the maximum unrelated set (up to third degree).^49^

### Genome-wide association analysis

Using the transformed speech rhythm perception phenotype (see study design), ∼7 million imputed variants were analyzed for their association with prosody perception scores in PLINK2^48^ using a linear regression model through the --glm command. Model covariates included age, sex, and the first five PCs explaining population substructure variation (based on the number of ancestry PCs that were correlated with the phenotype). Since GWAS model covariates were the same ones included during the phenotype transformation step (see study design) based on guidance in Sofer et al.,^55^ we also conducted the GWAS without the covariates to report results without the two-stage procedure (see supplemental information). Sentinel variants were defined as the most significant variant found within a ±1 MB window, and surpassed a minor allele count of 30.

### Annotation

We annotated genome-wide significant sentinel variants *(p* < 5.00 × 10^−8^) and suggestive significant signals (*p* < 5.00 × 10^−6^) using the Open Targets Genetics Variant-to-Gene (V2G) pipeline, which aggregates evidence from chromatin interactions, molecular quantitative trait loci, *in silico* functional predictions, and distance between the variant with the gene transcription start site.^56^^,^^57^ All reported positional coordinates (chromosome and base pair locations) refer to human genome reference build 37.

### Gene-based GWAS

Prior to performing our gene-based association analysis, we first annotated our autosomal SNPs to protein coding genes using the --annot command in MAGMA (v.1.09)^58^ and specifying a SNP annotation window of +35/−10kb.^59^ Next, we performed gene-based association analysis using MAGMA (v.1.09)^58^ on our individual-level genetic data and speech rhythm perception scores, controlling for age, sex, and first five PCs. Genome-wide significance was determined using a *p* value threshold < 2.71 × 10^−6^, a Bonferroni correction for the 18,441 genes tested.

### Gene set enrichment analysis of birdsong gene sets

We collated seven birdsong gene sets that were generated from microarray studies of zebra finch to find genes differentially expressed in association with singing behaviors.^60^ Set 3: singing vs. silence (Area X); set 5: singing vs. silence (robust nucleus of the arcopallium “RA”); set 6: singing vs. silence (the lateral magnocellular nucleus of the anterior nidopallium “LMAN”); set 7: singing vs. silence (Area X: controlling for differential expression in genes in the ventral striato-palladium “Ctrl VSP,” a non-singing-specific network in songbird brains); set 8: number of motifs sung (Area X: Ctrl VSP); set 10: listening/playback (two auditory areas, caudal medial nidopallium and the L2a portion of Field L, in males that heard song vs. silence). See Gordon et al.^60^ for details on gene set creation and access to curated gene sets. We then tested the enrichment of these gene sets in human speech rhythm perception using MAGMA (v.1.09).^58^ The birdsong gene sets tested were limited to sets containing more than 20 genes. Since Area X is a known ortholog of the basal ganglia,^61^^,^^62^ we created an “Area X overlap gene set,” which was the intersection of the 477 overlapping genes across all Area X birdsong gene sets (i.e., set 3: singing vs. silence [Area X]; set 7: singing vs. silence [Area X Ctrl VSP]; and set 8: number of motifs sung [Area X Ctrl VSP]). Gene set analysis significance was determined to be *p* < .0125, a Bonferroni correction for the four distinct brain regions of these gene sets (Area X: sets 3, 7, 8, and overlap; LMAN: set 6; RA: set 5; auditory areas: set 10). For gene set enrichment results with unadjusted *p* values < .05, we performed conditional gene set analysis conditioning on average gene expression^63^ in the brain^64^ (see supplemental information).

### PGS construction and testing

PGS models were trained using prior GWAS results for our three traits of interest: (1) word reading^2^ (*n* = 27,180), (2) beat synchronization^44^ (*n* = 606,825), and (3) voice pitch variability in reading^3^ (*n* = 12,901) using PRS-CS^65^. PRS-CS is a Bayesian regression method that places a continuous shrinkage prior on individual SNP weights for LD and variant significance. PGS models were trained using European genetic ancestry GWAS summary statistics. A European LD reference constructed from 1000 Genomes Project phase 3 was used for all analyses. Default auto-phi parameters were used to prevent model overfitting. The speech rhythm perception testing set (*n* = 1,698 individuals from all genetic ancestries, “multi-ancestry sample”) was scored using PLINK v.1.9.^48^

Similar to methods used in Gustavson et al.,^47^ PGSs were *Z* scored within each of the five genetic ancestry groups to reflect standardized associations between our trait of interest and speech rhythm perception scores. To determine if the genetic predisposition of our trait of interest predicted speech rhythm perception scores, we performed regression models where the primary outcome was speech rhythm perception scores regressed on the within-ancestry *Z* scored PGS scores, controlling for age, sex, and the first five PCs. All measures in the model were standardized. Our analyses focused on using the full multi-ancestry sample (*n* = 1,698). We also present analyses restricted to only individuals of European genetic ancestry (*n* = 1,501) in the supplemental information. Table 1 shows the sample sizes and age distributions for genetic ancestry groups.

**Table 1:** Demographic information and overview of speech rhythm perception performance

Genetic ancestry	*n* (% female)	Age (SD)	Raw % TOPsy scores (SD)	Median raw % TOPsy scores (range)
All	1,698 (74.17%)	45.11 (16.26)	76.75 (22.85)	84.38 (16.25, 100)
African	90 (90.00%)	43.42 (14.42)		
Hispanic/Latino	33 (69.70%)	34.09 (13.51)		
East Asian	49 (77.55%)	34.92 (12.72)		
South Asian	25 (52.00%)	31.20 (9.07)		
European	1,501 (73.55%)	46.02 (16.32)	77.68 (22.57)	86.25 (16.25, 100)

Genetic ancestry was derived by PC analysis. Due to ceiling effects, a linear model for TOPsy scores was fit adjusting for age, sex, and first five ancestry PCs. The residuals of the model were inverse ranked transformed and used as the speech rhythm perception phenotype (see subjects and methods).

## Results

### Speech rhythm perception TOPsy scores

To avoid ceiling effects, speech rhythm perception scores were transformed. A linear model for TOPsy scores was fit adjusting for age, sex, and first five ancestry PCs, and the inverse-rank transformed residuals of the model was used as the speech rhythm perception phenotype (see study design, Table 1). TOPsy scores were available for *n* = 1,729 people who also had clean genotyped data. Thirteen individuals were removed due admixture, 13 were removed due to relatedness (see subjects and methods), and 5 were dropped from models due to missing covariate information (i.e., age or sex). Hence, our total sample size was *n* = 1,698. Further phenotypic analyses of TOPsy scores in a larger overall sample which includes individuals without genotyping (*n* = 2,508), have been previously detailed.^41^

### Genome-wide association analysis

**Figure 1:**
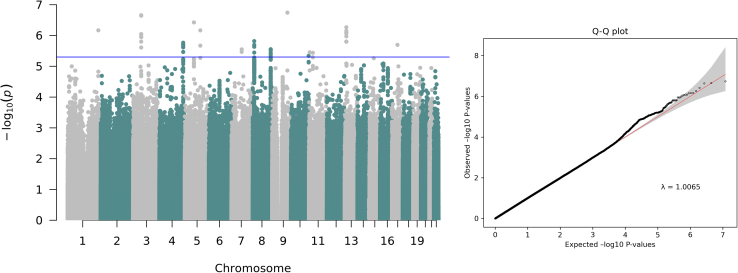
Manhattan plot and Q-Q plot for genome-wide association analysis of speech rhythm perception Genome-wide association analysis included 1,501 individuals of European genetic ancestry and 6,778,702 variants. Results showed 14 loci of interest at a suggestive significance *p* value threshold < 5.00 × 10^−6^ (indicated by the blue line). Q-Q plot *x* axis represents expected log_10_*p* and the *y* axis represents observed –log_10_*p*.

We performed a GWAS of speech rhythm perception, where our phenotype was based on performance on the TOPsy task.^41^ GWAS was carried out in 1,501 unrelated individuals of European genetic ancestry with ∼7 million variants tested (Table 1). The genomic inflation factor, λ, was 1.0065 (Figure 1). Although no variants surpassed genome-wide significance (*p* < 5.00 × 10^−8^), 14 loci reached suggestive significance (*p* < 5.00 × 10^−6^, Table 2). In supplementary analyses, we mapped these 14 signals to genes using the Open Targets V2G pipeline^53^^,^^54^ and queried these genes within the GWAS Catalog.^66^ We found that our speech rhythm perception-associated genes were previously associated with the following broad trait categories: Cardiac/Circulatory, Obesity/Endocrine/Metabolic, Metabolites, Neurological, and Mental Disorders (see supplemental information). Since the GWAS model covariates were included during the phenotype transformation step^55^ (see study design), we further performed the GWAS without covariates (see supplemental information, Table S2; Figure S2).

**Table 2:** Suggestive significant signals associated with speech rhythm perception

rsid	CHR	POS_b37	BETA	EA	NEA	EAF	SE	Functional gene(s)	*p* value
rs146064482	9	114369425	−0.719	T	G	0.018	0.137	*GNG10*	1.81E−07
rs56702966	3	60958999	0.301	C	T	0.11	0.058	*FHIT*	2.19E−07
rs6886492	5	68425699	−0.235	G	A	0.188	0.046	*SLC30A5*	3.75E−07
rs3000634	13	31269282	0.314	G	A	0.095	0.062	*ALOX5AP*	5.41E−07
rs80288146	1	231061747	0.597	T	C	0.024	0.12	*TTC13*	6.79E−07
rs67704630	5	116506760	0.401	C	T	0.054	0.08	NA	6.81E−07
rs11250130	8	11214455	0.178	A	G	0.473	0.037	*FAM167A*	1.52E−06
rs6837755	4	178680187	−0.183	A	G	0.325	0.038	*AGA*	1.71E−06
rs138312553	17	41023343	0.593	G	A	0.021	0.124	*AOC3*	2.01E−06
rs34483201	8	135010378	−0.187	T	C	0.3	0.04	*ST3GAL1*	2.79E−06
rs139396308	7	76384707	−0.746	A	G	0.013	0.159	*POMZP3*	2.81E−06
rs6578580	11	5214116	−0.174	C	T	0.424	0.037	*OR51V1*	3.51E−06
rs192407100	11	29370196	−0.536	T	C	0.026	0.115	*BDNF*	3.64E−06
rs12774548	10	132984505	−0.26	T	G	0.117	0.057	*TCERG1L*	4.57E−06

All signals presented are identified as the most significant variant found within a ±1 MB window (*p* < 5.00 x 10^-6^*)*. rsid, SNP; CHR, chromosome; POS_b37, position in hg37; BETA, the effect; EA, effect allele; NEA, non-effect allele; EAF, effect allele frequency; SE, standard error; functional gene(s), most likely implicated functional gene that was mapped using Open Targets Genetics; *p* value, association *p* value.

### Gene-based GWAS

**Figure 2:**
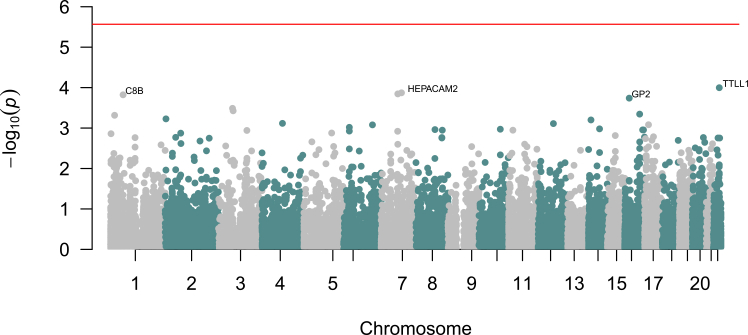
Gene-based genome-wide association analysis of speech rhythm perception Gene-based genome-wide association analysis included 1,501 individuals of European genetic ancestry and 18,441 genes. The red line indicates Bonferroni significance, *p* < 2.71 × 10^−6^, 0.05/18,441 genes tested.

To better prioritize genes associated with speech rhythm perception, we conducted a gene-based GWAS using MAGMA (v.1.09).^58^ Although our gene-based GWAS did not yield any genes surpassing Bonferroni significance (*p* < 2.71 × 10^−6^), top associated genes consisted of *TTLL1*, *GP2*, *C8B*, and *HEPACAM2*, *p* < 1.42 × 10^−4^ (Figure 2).

### Gene set analysis

**Figure 3:**
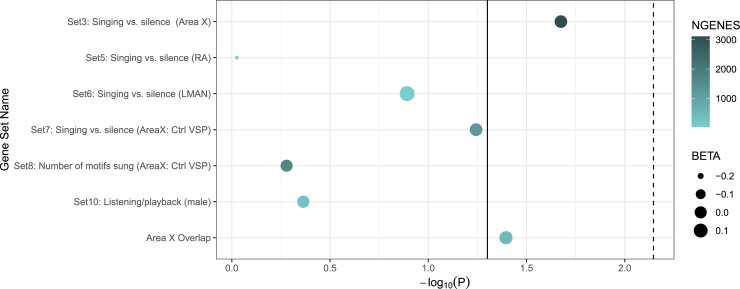
Birdsong gene set enrichment analysis of speech rhythm perception Gene sets were obtained from Gordon et al. 2021^60^. The solid line represents nominal significance, *p* < .05. The dotted line represents the Bonferroni gene set analysis significance, *p* < .0125, a Bonferroni correction for the four distinct brain regions sampled in the birdsong gene sets. Area X, a known ortholog of the human basal ganglia; Ctrl ventral striato-pallidum (“Ctrl VSP”), controlling for the differential expression of genes in a brain region involved in non-vocal motor function); LMAN, the lateral magnocellular nucleus of the anterior nidopallium; RA, robust nucleus of the arcopallium.

To explore the potential for evolutionary convergence between speech rhythm perception and other vocal learning behaviors, we performed gene set enrichment analysis using sets of genes that were differentially expressed in association with singing behaviors in zebra finches (see Gordon et al.^60^ for more details). Human speech rhythm perception was enriched for singing vs. silence in Area X and the Area X overlap gene set, *p* < .05 (Figure 3). No gene sets surpassed Bonferroni correction (*p* < .0125, a correction for the four brain regions spanning the birdsong gene sets (**Area X**: sets 3, 7, 8, and overlap; **LMAN**: set 6; **RA**: set 5; **auditory areas**: set 10). See Table 3 for full gene set enrichment analysis results. Conditional gene set analysis^63^ results controlling for average brain expression^64^ as a gene property can be found in Table S3.

**Table 3:** Gene set analysis for speech rhythm perception

Gene set	NGENES	BETA	SD	SE	*p* value
Set 3: Singing vs. silence (Area X)	3112	0.028	0.011	0.014	0.021
Area X overlap	462	0.058	0.009	0.033	0.040
Set 7: Singing vs. silence (Area X: Ctrl VSP)	1204	0.034	0.008	0.021	0.057
Set 6: Singing vs. silence (LMAN)	24	0.170	0.006	0.149	0.128
Set 10: Listening/playback (male)	261	0.007	0.001	0.042	0.433
Set 8: Number of motifs sung (Area X: Ctrl VSP)	1670	−0.001	0.000	0.019	0.526
Set 5: Singing vs. silence (RA)	26	−0.216	−0.008	0.137	0.942

### PGS analysis of word reading

**Figure 4:**
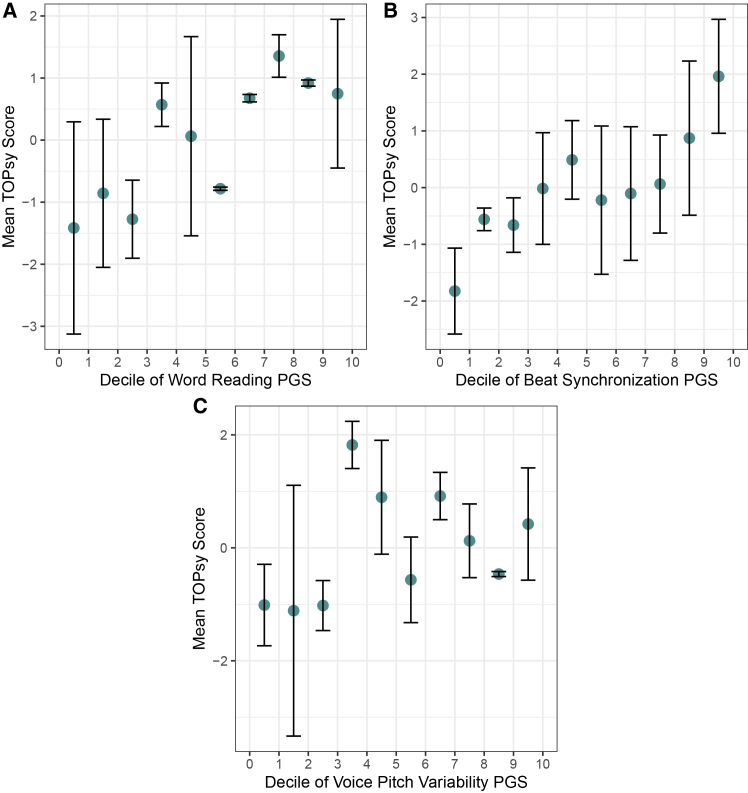
Polygenic associations between speech rhythm perception and other musicality and language traits Decile plots showing polygenic scores of (A) word reading, (B) beat synchronization, and (C) voice pitch variability on the *x* axis, and behavioral speech rhythm perception scores on the *y* axis, in *n* = 1,698. Polygenic scores were derived from GWAS results reported in Eising et al.,^2^ Niarchou et al.,^44^ and Gisladottir et al.,^3^ respectively. Polygenic scores and behavioral scores with standard error bars are visualized for *n* = 1,698 individuals from the full sample.

Speech rhythm perception and reading abilities are robustly phenotypically correlated in both children^16^^,^^17^^,^^67^^,^^68^^,^^69^ and adults,^19^^,^^46^ with prosody being recently highlighted as a missing factor in current models of reading and literacy development.^19^ To examine if there are relevant biological signals connecting speech rhythm perception and reading, we sought to determine if genetic predispositions for word reading abilities explain variability in speech rhythm perception scores. Word reading PGSs were derived from word reading summary statistics from the GenLang consortium.^2^ Genetic predispositions for word reading were positively correlated with speech rhythm perception phenotypes (β = 0.109; *p* = 2.23 × 10^−5^; 95% CI, 0.060, 0.160; *n* = 1,698; Figure 4A), after controlling for age, sex, and the first five ancestry PCs. These findings were replicated when tested only in individuals of European genetic ancestry (see supplemental information, Table S4; Figure S3A). Further comparison of word reading and speech rhythm perception GWAS results through concordance analysis can be found in Table S5.

### PGS analysis of beat synchronization

Musical rhythm and speech rhythm perception are also known to be phenotypically correlated; with rhythm and language traits (including prosody perception) being theorized to have underlying shared genetic architecture.^1^ To observe if a shared genetic etiology connects beat synchronization and speech rhythm perception, we tested if the genetic predisposition of beat synchronization predicts speech rhythm perception scores. Beat synchronization scores were based on a recent GWAS.^25^ We observed that the genetic predisposition of beat synchronization explains speech rhythm perception skills (β = 0.138; *p* = 1.11 × 10^−8^; 95% CI, 0.09, 0.18; *n* = 1,698; Figure 4B), after controlling for age, sex and, the first five PCs. These findings were replicated when testing in individuals of European genetic ancestry (see supplemental information, Table S4; Figure S3B). Further comparison of beat synchronization and speech rhythm perception GWAS results through concordance analysis can be found in Table S5.

### PGS analysis of voice pitch variability

Recent efforts have aimed to uncover the genetic architecture of voice pitch and vowel acoustics.^3^ Voice pitch variability in reading is phenotypically correlated with higher performance on verbal fluency tasks and reduced reading difficulties, and genetically correlated with education. To explore shared genetic signals connecting speech rhythm perception and voice pitch variability in speech production, both aspects of prosody perception and production, we tested if the genetic predisposition of voice pitch variability predicts speech rhythm perception scores. Voice pitch variability scores were obtained from a recent GWAS on voice pitch and vowel acoustics.^3^ We found that the genetic predisposition of voice pitch variability predicts speech rhythm perception skills (β = 0.062; *p* = 1.08 × 10^−2^; 95% CI, −0.428, 0.552; *n* = 1,698; Figure 4C), after controlling for age, sex, and the first five PCs. However, this finding did not replicate when testing only in individuals of European genetic ancestry (see supplemental information, Table S4; Figure S3C).

## Discussion

The current study performs a genome-wide investigation of speech rhythm perception (an aspect of speech prosody perception), which is an important yet often overlooked aspect of speech and language abilities. Although no loci surpassed genome-wide significance (*p* < 5.00 × 10^−8^), GWAS results revealed 14 signals reaching suggestive significance (*p* < 5.00 × 10^−6^). Results of polygenic score analyses also suggest a common genetic architecture connecting speech rhythm perception and language, reading, and musical rhythm (i.e., beat synchronization) traits. Further, we showed that human prosody perception shares genetic signatures with key vocal learning brain regions in songbirds, consistent with theories of evolutionary convergence of communication mechanisms across species.

Since our sample size was underpowered (Figure S4) and did not reveal any genome-wide signals, we further explored the 14 signals that reached suggestive significance to begin to map the biology of prosody and point to directions for future research. Mapping these 14 loci to genes and querying them in the GWAS Catalog^66^ revealed biological relevance related to the following broad trait categories: Cardiac/Circulatory, Obesity/Endocrine/Metabolic, Metabolites, Neurological, and Mental Disorders. These findings are particularly interesting given that impairments in prosody have been observed in various mental disorders and neurological conditions, such as autism spectrum disorder,^70^ depression,^71^ right-brain damage,^72^ and Alzheimer's disease.^73^ Although most prior studies investigating prosody and mental health or neurological disorders primarily focused on prosody *production*, our findings about prosody *perception* converge with these previous investigations, potentially highlighting its underestimated relevance for mental and neurological disorders. Prosody perception is therefore an area ripe for future study in the context of mental health, psychiatric, and neurological domains, among others.

Individual-level cross-trait PGS analyses offer additional validation of, and confidence in, the speech rhythm perception phenotype (i.e., TOPsy scores). As predicted, musical rhythm and reading traits—both known to be phenotypically correlated with prosody perception—also exhibited relevant biological connections with it. Specifically, genetic predispositions for word reading abilities predicted speech rhythm perception scores in an independent sample. These results reveal biological evidence consistent with long-standing findings about the importance of prosody perception in reading outcomes across the lifespan.^16^^,^^17^^,^^19^^,^^46^^,^^67^^,^^68^^,^^69^^,^^74^^,^^75^ Similarly, musical rhythm skills are a known behavioral and neural correlate of speech rhythm abilities^39^^,^^42^^,^^43^^,^^76^ and were also genetically associated in PGS analyses, consistent with the Musical Abilities, Pleiotropy, Language, and Environment (MAPLE) framework,^1^ which posits that shared genetic influences partially drive the widespread associations seen between several musicality and speech-language traits. Taken together, these findings reiterate the relevance of genetically influenced neural endophenotypes that may underlie individual differences in these intercorrelated traits (i.e., prosody, musical rhythm, reading).

Our gene-based GWAS revealed that *TTLL1* and *GP2* genes were in the top 5 most highly associated with speech rhythm perception, although no genes surpassed statistical thresholds for genome-wide significance. *TTLL1* is involved in microtubule organization,^77^ which in turn is important for cellular morphology and function, including those involved in neurodevelopmental, degeneration, and regeneration processes.^78^ Additionally, G*P2* has been associated with sleep quality^79^ and endocrine-related traits.^80^^,^^81^ *GP2*’s association with sleep quality is particularly interesting given that the genetic architecture of beat synchronization, capturing an aspect of musical rhythm perception, was correlated with other biological-rhythm traits, including circadian chronotype and insomnia.^44^ These findings begin to shed light on the potential biological mechanisms involved in speech rhythm perception, and should be validated in future well-powered samples.

Cross-species comparisons further shed light on the potential evolutionary convergence of human speech rhythm perception and vocal learning traits in other taxa. Genes associated with our prosody perception phenotype were enriched for genes expressed in Area X of the songbird brain during birdsong. Area X is a known ortholog for the human basal ganglia,^61^^,^^62^ which has been previously studied for its relevance to musical rhythm abilities and neural processing.^82^^,^^83^^,^^84^^,^^85^^,^^86^ Relatedly, previous studies have shown that genes associated with human musical beat synchronization (i.e., musical rhythm abilities) are enriched for genes expressed during birdsong,^60^ complementing genetic and neural similarities in the cortico-basal-ganglia circuitry across species.^33^ These results demonstrate fundamental connections between prosody perception in humans and other vocal learning species and are consistent with theories of language evolution. Current theories posit that early prosody-rich protolanguage sounds facilitated survival and led to the emergence of complex language (i.e., syntax).^25^ Further, facets of human speech and language abilities are thought to have convergently evolved from pre-adaptations in vocal learning species more broadly.^30^^,^^31^

Some limitations of the study include small sample size and overrepresentation of individuals of European genetic ancestry (∼88% of our sample). Notably, our sample size lacked the sufficient power (Figure S4) to identify speech rhythm perception-associated variants (especially since common genetic variants have small to moderate effects), and further reliably estimate SNP-based heritability. There are also limitations to GWAS more generally, including lack of power to detect modest and low effects at rare variants, challenges related to the quality and specificity of phenotypes, inability to distinguish protective from deleterious effects, and frequent overrepresentation of individuals of European genetic ancestry. Increased sample size and diversity can be achieved by deploying the TOPsy task and/or including questions pertaining to speech and rhythm within large biobank studies. Indeed, the TOPsy task’s design enables large-scale rapid phenotyping and can facilitate future genomics and epidemiological efforts, as well as more targeted investigations of prosody perception in psychiatric and neurological disorder contexts. Further, since these genomic findings were captured from the same participants as those involved in the validation of the phenotyping tool TOPsy,^41^ future research should replicate phenotypic and genomic efforts related to prosody perception to ensure rigor and external validations. Given the paucity of genetics studies of speech rhythm perception, these initial results are promising and should serve as potential avenues for future validation and investigation of other language and reading traits. Additionally, it is important to highlight that, although prosody is typically phenotypically linked to phonological processing skills, the manifestation of prosodic skills is a multi-dimensional phenomenon encompassing a range of acoustic features, communicative functions, and cognitive processes. The complexity of prosody can potentially explain why our analyses linking speech rhythm perception to PGSs of another prosody phenotype (i.e., voice pitch variability while reading) were inconclusive. Indeed, both of these constructs were measuring different components of prosody, emphasizing the need for diverse and nuanced phenotyping and well-powered study designs.

This study represents a foundational step in understanding the biology relating to individual differences in prosody perception. Our findings lay the groundwork for further exploration into the genetic architecture of speech-language traits; genetic epidemiology linking speech and language function to health more broadly; and educationally relevant outcomes such as reading development and adult literacy. These future directions could improve precision efforts in both communication sciences and disorders and educational settings.

## Data and code availability

• For the subset of individuals with speech rhythm perception scores, genotype data for the Vanderbilt Online Musicality Study will be made available to qualified investigators in dbGaP.
• Speech rhythm perception summary statistics genome-wide are available publicly through the GWAS Catalog. The accession number for the speech rhythm perception summary statistics is GWAS Catalog: GCST90809464.
• GWAS summary statistics are available publicly for word reading through the GWAS Catalog (GWAS Catalog: GCST90104463) or through the GenLang network website (see web resources).
• GWAS summary statistics for voice pitch variability and beat synchronization are restricted based on data use agreements.

## Acknowledgments

We acknowledge feedback from these additional colleagues, who served as members of our project advisory board: Drs. Matthew Leonard, Duane Watson, and Jennifer Zuk. We would like to thank participants from 23andMe and the Vanderbilt Online Musicality Study. This work was supported by funding from the 10.13039/100000001National Science Foundation (NSF 1926794 and NSF 1926736) and by funding from the 10.13039/100000055National Institute on Deafness and Other Communication Disorders (NIDCD), Office of the Director (OD), and National Institute on Drug Abuse (NIDA), of the 10.13039/100000002National Institutes of Health (NIH), under award numbers R01DC016977, R01DC017175, R01DA059804, R21DC021276, R03DC021550, and F31DC022482. Data collection and management via REDCap was made possible through awards from the 10.13039/100006108NCATS/10.13039/100000002NIH (UL1 TR000445). The content is solely the responsibility of the authors and does not necessarily represent the official views of the funders.

## Author contributions

R.L.G. and S.N. oversaw study; R.L.G. designed the study, with conceptual contributions from S.N., N.C., J.E.B., N.J.C, and R.S.G; A.C.S., S.N., and R.L.G. drafted and revised the manuscript, with contributions from J.E.B., L.E.P., H.M.H., N.C., D.E.G., R.S.G., and C.L.M; A.C.S. and S.N. performed analyses, with contributions from P.L.C., T.L.H., and X.L.D; Y.W. and D.E.G. oversaw data management; P.L.C. performed quality control on the genetic data. All authors critically reviewed the manuscript.

## Declaration of interests

The authors declare no competing interests.

## Web resources

GenLang network, https://www.genlang.org/

GWAS Catalog, https://www.ebi.ac.uk/gwas/

Open Targets Genetics, https://platform.opentargets.org/

## Supplemental information

Document S1. Figures S1–S4, Tables S2–S4, and Method S1

Table S1. Speech rhythm perception-associated genes within the GWAS Catalog

Document S2. Article plus supplemental information
