# Supplementary material for: Genome-wide investigation of prosody perception: Shared genetic influences between speech rhythm, musical rhythm, and reading traits
Source: HGG Adv. 2026 Feb 18;7(3):100581. doi: 10.1016/j.xhgg.2026.100581 (PMC13091348; doi:10.1016/j.xhgg.2026.100581)
Supplement: Document S1. Figures S1–S4, Tables S2–S4, and Method S1 [file mmc1.pdf]

HGGA, Volume 7

Supplemental information

Genome-wide investigation of prosody perception: Shared genetic influences between speech rhythm, musical rhythm, and reading traits

Alyssa C. Scartozzi, Youjia Wang, Peyton L. Coleman, Ximena León Du'Mottuchi, Tara L. Henechowicz, Daniel E. Gustavson, Lauren E. Petty, Heather M. Highland, Nicole Creanza, Cyrille L. Magne, Rosa S. Gísladóttir, Nancy J. Cox, Jennifer E. Below, Srishti Nayak, and Reyna L. Gordon

Supplemental Information

Supplemental Methods

Genome-wide association analysis of speech rhythm perception without the inclusion of covariates in the linear model

Since GWAS model covariates were used during our phenotype transformation step, we ran an additional GWAS without the covariates. A GWAS was performed with the transformed prosody perception scores in PLINK2[1] using a linear regression model using the flag --glm allow-no-covar. Sentinel variants were identified as the most significant variant within a +/- MB window with a minor allele count greater than 30.

Biological associations with speech rhythm perception genes in GWAS Catalog

To better characterize our genome-wide association loci, we queried our top signals (*p*-value < 5.00×10^{-6}) within the GWAS Catalog.[2] First, we filtered the GWAS Catalog (release date: 2024-03-01) to only include results that surpassed genome-wide significance, *p*-value < 5.00×10^{-8}. Next, we used our speech rhythm perception genome-wide significant and suggestive genes that were mapped using the Open Targets Genetics Variant-to-Gene (V2G) pipeline,[3,4] Table 2) in our GWAS Catalog search. Trait associations for these 13 genes were then manually categorized into 21 trait categories (Table S1a), where only 11 of our prosody-associated genes were found within the GWAS Catalog. Our speech rhythm perception findings and their associations

within the GWAS Catalog can be found in Table S1b. The number of unique speech rhythm perception genes found within each of the 20 trait categories can be found in Figure S1.

Figure S1. Unique number of speech rhythm perception-associated genes found within GWAS Catalog trait categories. Human body figure showing the number of unique speech rhythm perception-associated genes from our GWAS analysis associated with manually categorized broad trait categories in the GWAS Catalog (release date: 2024-03-01). Eleven of the suggestive signals (p-value < 5.00 x 10^{-6}) were found within the GWAS Catalog. Trait categories and GWAS traits can be found within Table S1.

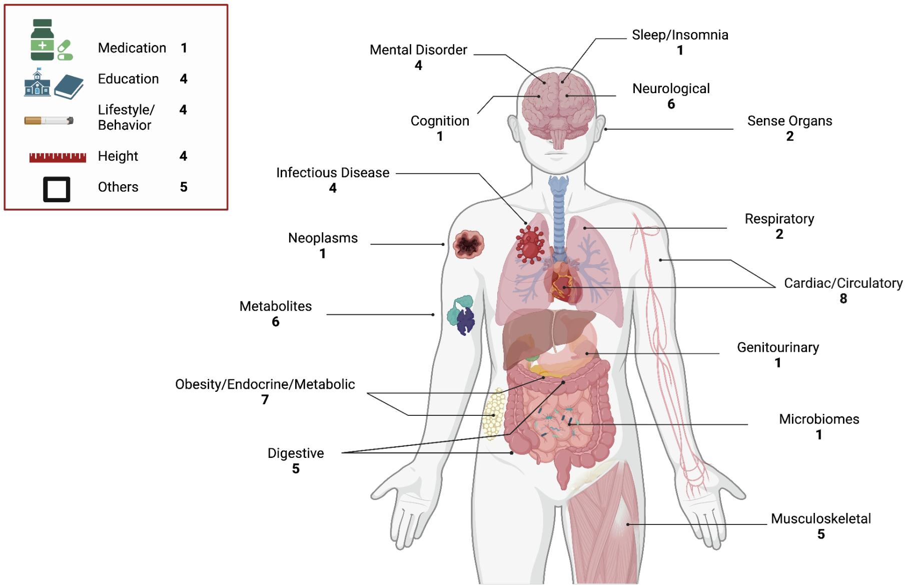

Conditional gene-set enrichment analysis of the birdsong gene sets in human speech rhythm perception

We performed the conditional gene-set enrichment analysis in MAGMA (v.1.09).[5] For our conditional gene-set enrichment analysis, average brain expression values from GTEx v8[6] was used as a gene property covariate for the birdsong gene sets with unadjusted p-values < 0.05, Singing versus Silence (Area X) and the Area X overlap.

PGS analysis in individuals of European genetic ancestry

Since our discovery GWASs were performed in individuals of European genetic ancestry, we performed PGS analyses restricting the speech rhythm perception dataset to individuals of European genetic ancestry (N = 1,501). All analyses used the European LD reference from 1000 Genomes Project Phase 3, with default auto-phi settings.

Concordance analysis with discovery GWAS and speech rhythm perception GWAS results

We performed concordance analyses to assess if there is increased directional consistency in overlapping SNPs between our speech rhythm perception GWAS and our discovery GWAS that demonstrated significant PGS, word reading[7] and beat synchronization.[8] Concordance analyses were performed in LD pruned subsets of SNPs using the following *p*-value thresholds between both sets of summary statistics: 0.05, 0.005, and 0.0005. LD pruning was performed using the plink[1] --indep-pairwise command with a window size of 10kb, step size of 1, and r2 threshold of 0.2. For a given *p*-value threshold, this analysis defines the concordance rate as the number of genetic variants with the same direction of effect divided by the total number of shared genetic variants between the two datasets. A one-sample t-test was performed to determine the concordance rate was significantly higher than expected by chance. For more information on implementation of this method see [9,10].

GWAS Power Calculations

To compute quantitative trait GWAS power, we leveraged the R package gwas-power.[11] We used the power_beta_maf function using a sample size of 1,501, beta value of 0.10 to 0.50, minor allele frequency 0.05 to 0.25, and *p*-value of 5.00 x 10^{-8}.

Supplemental Results

Genome-wide association analysis without covariates

A genome-wide association on speech rhythm perception was performed without using covariates in our linear model in 1,501 unrelated individuals of European genetic ancestry. The genomic inflation factor, λ, was 1.0117 (Figure S2). No variants surpassed genome-wide significance (*p*-value < 5.00 x 10^{-8}) and 13 loci reached suggestive significance (*p*-value < 5.00 x 10^{-6}, Table S2).

Figure S2. Manhattan plot and Q-Q plot for genome-wide association analysis of speech rhythm perception without including covariates in the linear model. Genome-wide association analysis included 1,501 individuals of European genetic ancestry and 6,778,702 variants. Genome-wide significance *p*-value 5.00 x 10^{-8} is indicated by the red line. Results showed thirteen loci of interest at a suggestive significance *p*-value threshold < 5.00 x 10^{-6} (indicated by the blue line). Q-Q plot x axis represents expected $\log_{10} p$ and the y axis represents observed $-\log_{10} p$.

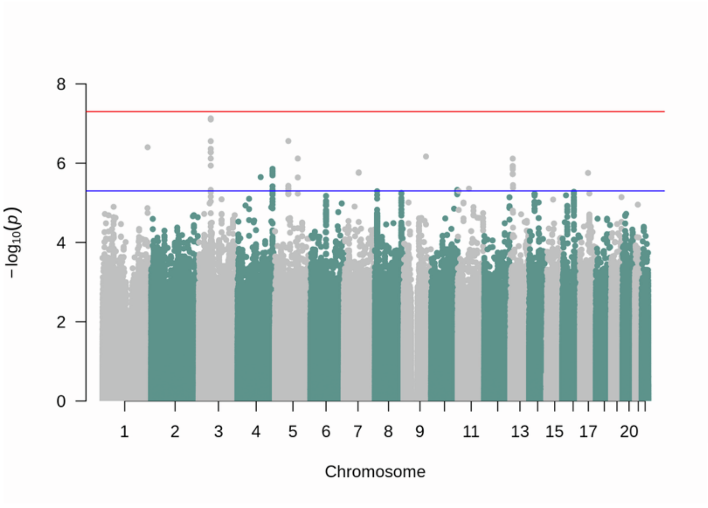

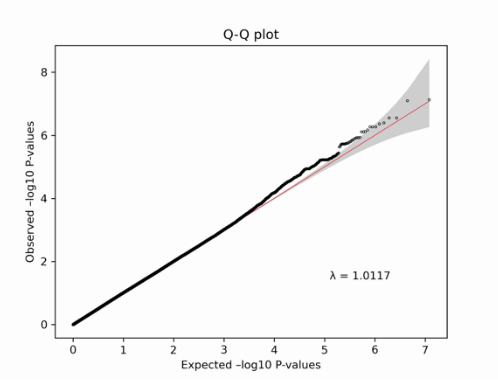

Table S2. Suggestive significant signals

associated with speech rhythm perception without including covariates in our linear model. All signals presented are identified as the most significant variant found within a +/- 1 MB window. ‘Rsid’: SNP, ‘CHR’: chromosome, ‘POS_b37’: position in hg37, ‘BETA’: the effect, ‘EA’: effect allele, ‘NEA’: non-effect allele, ‘EAF’: effect allele frequency, ‘SE’: standard error, ‘Functional Gene(s)’: most likely implicated functional gene that was mapped using Open Targets Genetics, ‘*p*-value’: association *p*-value.

rsid	CHR	POS_b37	BETA	EA	NEA	EAF	SE	Functional Gene(s)	*p*-value
rs56702966	3	60958999	0.313	C	T	0.110	0.058	*FHIT*	7.41E-08
rs6886492	5	68425699	-0.238	G	A	0.188	0.046	*SLC30A5*	2.77E-07
rs80288146	1	231061747	0.610	T	C	0.024	0.120	*TTC13*	3.97E-07
rs146064482	9	114369425	-0.686	T	G	0.018	0.137	*GNG10*	6.79E-07
rs67704630	5	116506760	0.400	C	T	0.054	0.081	NA	7.64E-07
rs3000634	13	31269282	0.311	G	A	0.095	0.063	*ALOX5AP*	7.69E-07
rs6837755	4	178680187	-0.186	A	G	0.325	0.038	*AGA*	1.39E-06
rs139396308	7	76384707	-0.764	A	G	0.013	0.159	*POMZP3*	1.71E-06
rs138312553	17	41023343	0.599	G	A	0.021	0.125	*AOC3*	1.77E-06
rs146770027	4	118198184	0.856	C	A	0.010	0.180	NA	2.25E-06
rs798971	13	32758834	0.192	A	G	0.261	0.041	*NAA50P1*	3.57E-06
rs150928534	11	57464253	0.613	A	G	0.019	0.133	NA	4.39E-06
rs12774548	10	132984505	-0.260	T	G	0.117	0.057	*TCERG1L*	4.74E-06

Speech rhythm perception associated genes found within the GWAS Catalog

From the 14 suggestive significant signals within our speech rhythm perception GWAS (*p*-value < 5 x 10^{-6}), 13 genes were mapped via the Open Targets Genetics V2G pipeline[3,4] (Table 2). To assess which trait categories may be associated with our speech rhythm perception-associated genes, we queried the GWAS Catalog[2] (Table S1 and Figure S1). From the 13 speech rhythm perception-associated genes, only 11 were found within the GWAS Catalog. We found that eight speech rhythm perception genes were found to be previously associated with Cardiac/Circulatory traits, seven with Obesity/Endocrine/Metabolic traits, and six with Metabolic traits. We further found that

six speech rhythm perception genes were found to be previously associated with Neurological traits, and four with Mental Disorders, with three genes overlapping between these two categories. GWAS trait categorization can be found within Table S1a. The full results for the number of unique speech rhythm perception-associated genes with trait categories can be found within Figure S1 and Table S1b.

Gene-set enrichment analysis controlling for average brain expression

Results for the conditional gene-set analysis,[5] which used average brain expression as a gene property covariate, show that human speech rhythm perception's enrichments singing vs silence in Area X and the Area X overlap gene sets remain nominally significant when controlling for average brain expression levels as a gene property, *p*-value < 0.05 (Table S3). These results do not surpass Bonferroni correction for the number of gene sets tested (7 gene sets).

Table S3. Conditional gene-set analysis on the two nominally significant gene-set analysis results. This follow-up analysis controlled for average brain expression as a gene property covariate. 'Gene Set': the gene-set being tested, 'NGENES': the number of genes found within each gene-set, 'BETA': the regression coefficient, 'BETA_STD': the predicted change in Z-value given a change of one standard deviation in the predictor gene-set, 'SE': the standard error of the regression coefficient, '*p*-value': the *p*-value.

Gene Set	NGENES	BETA	BETA STD	SE	*p*-value
Set 3: Singing vs. silence (Area X)	3090	0.026	0.010	0.014	0.033
Area X Overlap	460	0.056	0.009	0.033	0.048

PGS analysis of word reading, beat synchronization, and voice pitch variability in individuals of European genetic ancestry

To directly match the genetic ancestry to our discovery GWASs of word reading, beat synchronization and voice pitch variability, we performed PGS analyses predicting the genetic predisposition of our discovery GWAS trait on speech rhythm perception scores solely in individuals of European genetic ancestry (N = 1,501). We found that the genetic predisposition of word reading[7] and beat synchronization[8] explains speech rhythm perception scores (Table S3, Figures S2a and S2b). However, we found that the genetic predisposition of voice pitch variability[12] did not explain speech rhythm perception scores (Table S3, Figures S2c). All analyses controlled for age, sex, and the first five PCs.

Table S4. Speech rhythm perception skills predicted by polygenic scores for word reading, beat synchronization, and voice pitch variability in individuals of European Genetic Ancestry (N = 1,501). PGS testing controlled for age, sex, and the first five PCs. 'Polygenic Score': the discovery GWAS used to score speech rhythm perception individuals, 'β': effect, '95% CI': 95% confidence intervals, '*p*-value': polygenic score testing *p*-value.

Polygenic Score	β	95% CI	*p*-value
Word Reading	0.09791	[0.045, 0.150]	2.44×10^{-4}
Beat Synchronization	0.144	[0.096, 0.192]	5.91×10^{-9}
Voice Pitch Variability	0.03184	[-0.017, 0.080]	1.99×10^{-1}

Figure S3. Decile plots showing polygenic scores of (a) word reading, (b) beat synchronization, and (c) voice pitch variability on the x-axis, and behavioral speech rhythm perception scores on the y-axis, in N = 1,698. Polygenic scores were derived from GWAS results reported in Eising et al. (2022), Niarchou et al., (2022), and Gisladottir et al. (2023), respectively. Polygenic scores and behavioral scores with standard error bars are visualized for N = 1,501 individuals from European genetic ancestry.

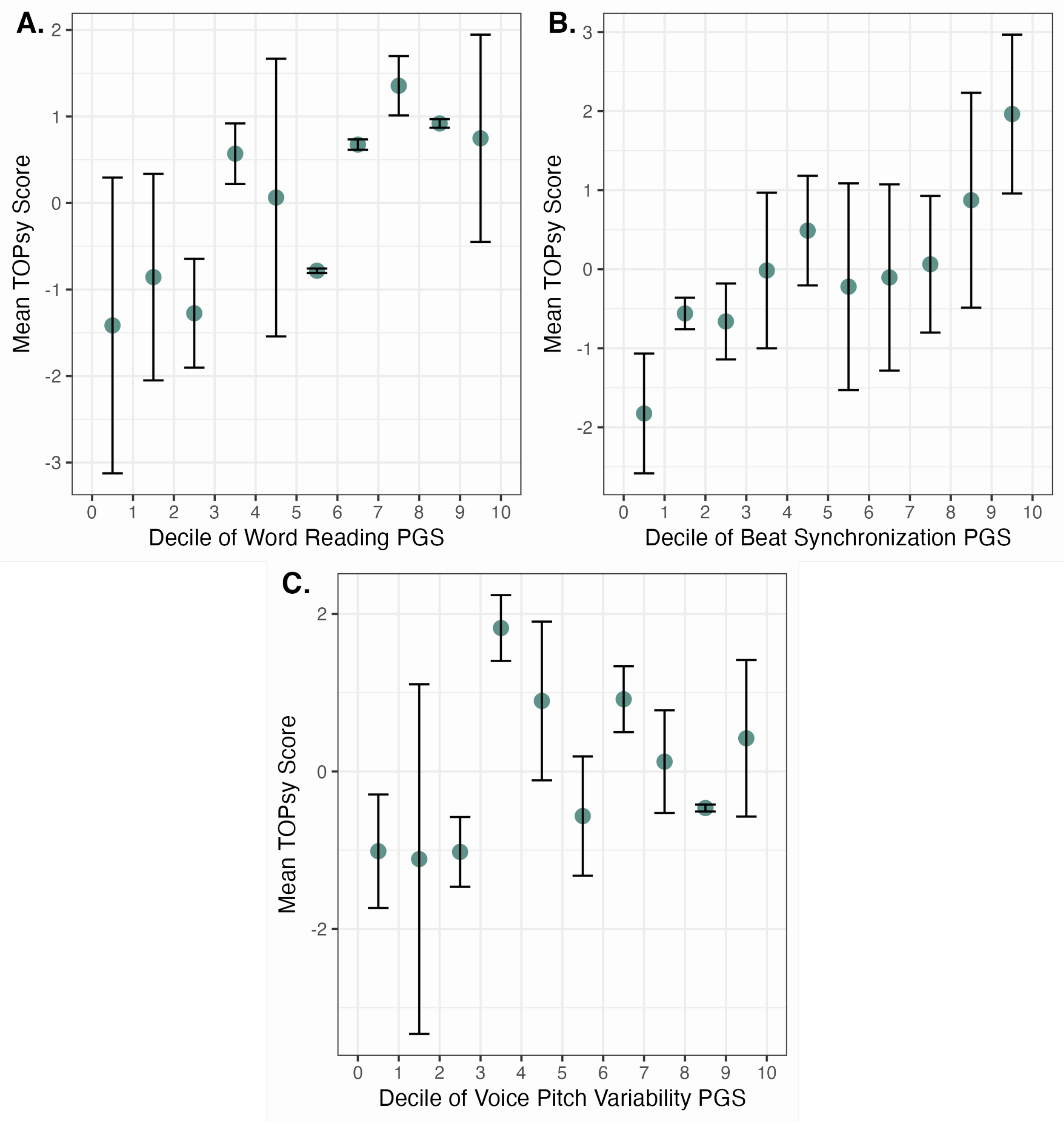

Concordance analysis results between our discovery GWAS and speech rhythm perception GWAS results

At the *p*-value threshold of 0.05 for beat synchronization and speech rhythm perception, we did observe significant concordance (concordance rate = 0.545, *p*-value = 3.857 x 10^{-14}, Table S5). However, our results do not demonstrate statistically

significant concordant effects at the other *p*-value thresholds for beat synchronization and speech rhythm perception. Further, we do not see statistically significant concordant effects for word reading and speech rhythm perception (Table S5).

Table S5. Concordance rates for speech rhythm perception and beat synchronization and speech rhythm perception and word reading. Concordance rate is defined as the number of overlapping LD pruned SNPs in the same direction divided by the total number of SNPs overlapping within a *p*-value threshold.

Comparison tested	*p*-value threshold	Total SNP overlap	Concordance rate	Concordance *p*-value
Speech rhythm perception and beat synchronization	0.05	6778	0.545	3.857×10^{-14}
	0.005	155	0.568	0.054
	0.0005	2	1.0	0.25
Speech rhythm perception and word reading	0.05	5154	0.505	0.222
	0.005	71	0.549	0.238
	0.0005	0	-	-

Power Calculation Results

Based on our power calculations, we are ~80% powered to detect a beta of 0.38 for variants with an MAF of 0.1, and a beta of 0.26 for variants with an MAF 0.25 (Figure S4).

Figure S4. Power calculation distribution by Minor Allele Frequency. X-axis represents Beta and y-axis represents power.

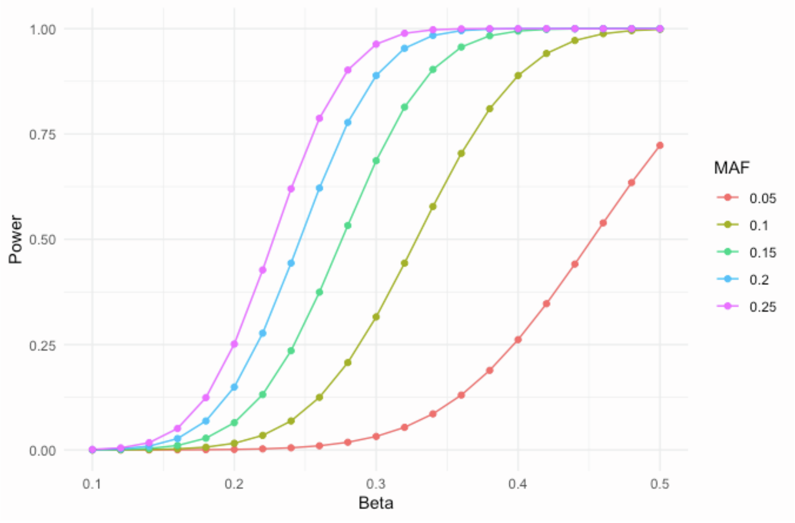

References

1. Purcell, S. *et al.* PLINK: A Tool Set for Whole-Genome Association and Population-Based Linkage Analyses. *Am. J. Hum. Genet.* **81**, 559–575 (2007).
2. Buniello, A. *et al.* The NHGRI-EBI GWAS Catalog of published genome-wide association studies, targeted arrays and summary statistics 2019. *Nucleic Acids Res.* **47**, D1005–D1012 (2019).
3. Mountjoy, E. *et al.* An open approach to systematically prioritize causal variants and genes at all published human GWAS trait-associated loci. *Nat. Genet.* **53**, 1527–1533 (2021).
4. Ghoussaini, M. *et al.* Open Targets Genetics: systematic identification of trait-associated genes using large-scale genetics and functional genomics. *Nucleic Acids Res.* **49**, D1311–D1320 (2021).
5. de Leeuw, C. A., Stringer, S., Dekkers, I. A., Heskes, T. & Posthuma, D. Conditional and interaction gene-set analysis reveals novel functional pathways for blood pressure. *Nat Commun* **9**, 3768 (2018).
6. Aguet, F. *et al.* The GTEx Consortium atlas of genetic regulatory effects across human tissues. *Science* **369**, 1318–1330 (2020).
7. Eising, E. *et al.* Genome-wide analyses of individual differences in quantitatively assessed reading- and language-related skills in up to 34,000 people. *Proc. Natl. Acad. Sci.* **119**, e2202764119 (2022).
8. Niarchou, M. *et al.* Genome-wide association study of musical beat synchronization demonstrates high polygenicity. *Nat. Hum. Behav.* **6**, 1292–1309 (2022).

9. Shaw, D. M. *et al.* Phenome risk classification enables phenotypic imputation and gene discovery in developmental stuttering. *Am. J. Hum. Genet.* **108**, 2271–2283 (2021).
10. Polikowsky, H. G. *et al.* Large-scale genome-wide analyses of stuttering. *Nat. Genet.* **57**, 1835–1847 (2025).
11. Visscher, P. M. *et al.* 10 Years of GWAS Discovery: Biology, Function, and Translation. *Am. J. Hum. Genet.* **101**, 5–22 (2017).
12. Gisladottir, R. S. *et al.* Sequence variants affecting voice pitch in humans. *Sci. Adv.* **9**, eabq2969 (2023).
