# Supplementary material for: Genome-wide investigation of prosody perception: Shared genetic influences between speech rhythm, musical rhythm, and reading traits
Source: HGG Adv. 2026 Feb 18;7(3):100581. doi: 10.1016/j.xhgg.2026.100581 (PMC13091348; doi:10.1016/j.xhgg.2026.100581)
Supplement: Document S2. Article plus supplemental information [file mmc3.pdf]

HGG
Advances

ARTICLE

Genome-wide investigation of prosody perception: Shared genetic influences between speech rhythm, musical rhythm, and reading traits

Alyssa C. Scartozzi,[1,11,*] Youjia Wang,[2,3] Peyton L. Coleman,[1,4] Ximena León Du'Mottuchi,[5] Tara L. Henechowicz,[6] Daniel E. Gustavson,[6] Lauren E. Petty,[1] Heather M. Highland,[7] Nicole Creanza,[5] Cyrille L. Magne,[8] Rosa S. Gísladóttir,[9] Nancy J. Cox,[1] Jennifer E. Below,[1] Srishti Nayak,[1,2,8,10,*] and Reyna L. Gordon[1,2,10]

Summary

Prosody perception is an often overlooked aspect of human language despite its importance in facilitating spoken language comprehension. Sensitivity to prosodic cues varies between individuals, and prosody perception skills are shown to be associated with various language- and reading-related outcomes. Despite the importance of prosody perception in human communication, its underlying biology is poorly understood. This study investigates the genetic architecture of prosody (speech rhythm) perception and explores its evolutionary roots. We conducted a GWAS of prosody ($n = 1{,}501$) as measured by scores on the Test of Prosody via Syllable Emphasis ("TOPsy"). GWAS results yielded 14 suggestive significant signals ($p < 5.00 \times 10^{-6}$). Gene set enrichment analysis identified shared genetic architecture between human prosody perception and key vocal learning brain regions in songbirds, suggesting that human prosody perception may have evolutionary convergence in communication mechanisms in animal vocal learning. Additionally, cross-trait polygenic score analyses suggest shared genetic influences between prosody perception and both word reading and musical beat synchronization, emphasizing how genetics influence prosody perception and its associations with communication-, education-, and music-related traits. These initial efforts could inform advances in communication sciences and disorders as well as educational contexts.

Introduction

Our capacity to communicate using spoken language, which we generally learn from a young age, is one of the most remarkable features of the human mind. While most research on language traits has focused on neuroscience and behavioral methods, there is emerging interest in the potential genetic influences on human language development and function. Twin- and family-based studies show that there are moderate to strong genetic influences on several speech and language traits, with heritability estimates ranging from 0.46 to 0.97 (see Nayak et al.[1] for a brief review). Although genetic investigations of language and disorders have primarily focused on family and linkage-type analyses, recent genome-wide association studies (GWASs) show that many speech, language, and reading-related traits and disorders (i.e., reading-related traits,[2] speech acoustics,[3] dyslexia,[4] and stuttering[5–7]) are moderately influenced by common genetic variation. For example, up to 13% of the variation in nonword repetition (a task characterized by listening and repeating back nonsense/meaningless words) is explained by differences in common genetic variants.[2] These studies suggest that language-related traits are polygenic, complex, and moderately distinct from other cognitive and educational traits,[8] with some degree of shared genetic architecture with other brain, health, and behavioral phenotypes.[9] Moreover, genetic variation plays a role in the development, structure, and function of the brain's language network.[2,9,10]

An often overlooked aspect of language is prosody, defined as the intonational and durational patterns within spoken language. Speech rhythm perception is a key aspect of prosody that involves the recognition of stress patterns in language. For example, the meaning of the sentence "John bought the car" changes depending on where the stress is placed (e.g., "*John* bought the car" [not Fred] or "John bought the *car*" [not motorcycle]). This intricate skill allows us to detect variations in emphasis within words, sentences, and larger spoken language structures. Sensitivity to spoken stress patterns emerges from infancy[11] and becomes particularly

[1]Vanderbilt Genetics Institute, Vanderbilt University Medical Center, Nashville, TN 37203, USA; [2]Department of Otolaryngology - Head & Neck Surgery, Vanderbilt University Medical Center, Nashville, TN 37232, USA; [3]Medical College of Wisconsin, Department of Internal Medicine and Pediatrics, Milwaukee, WI 53226, USA; [4]Center for Digital Genomic Medicine, Vanderbilt University Medical Center, Nashville, TN 27203, USA; [5]Department of Biological Sciences, Vanderbilt University, Nashville, TN 37203, USA; [6]Institute for Behavioral Genetics, University of Colorado Boulder, Boulder, CO 80303, USA; [7]Department of Epidemiology, UTHealth Houston, Houston, TX 77030, USA; [8]Department of Psychology, Middle Tennessee State University, Murfreesboro, TN 37132, USA; [9]Department of Icelandic and Comparative Cultural Studies, University of Iceland, Saemundargata 2, 102 Reykjavik, Iceland
[10]Senior author
[11]Lead contact
*Correspondence: alyssa.c.scartozzi@vanderbilt.edu (A.C.S.), srishti.nayak@vumc.org (S.N.)
https://doi.org/10.1016/j.xhgg.2026.100581.
© 2026 The Authors. Published by Elsevier Inc. on behalf of American Society of Human Genetics.
This is an open access article under the CC BY license (http://creativecommons.org/licenses/by/4.0/).

significant during mid-childhood and elementary education. During this period, children draw on speech rhythm cues to enhance crucial linguistic processes encompassing lexical retrieval,[12] morpho-syntactic parsing,[13] and understanding the intended meaning of a sentence beyond the literal words.[14] Additionally, speech rhythm perception skills are highly related to success in learning to read[15–18]; and its importance persists into adult literacy outcomes[19] and silent reading representations, where readers project prosodic changes onto text read silently ("Implicit Prosody Hypothesis").[20,21] Together, these studies highlight the importance of prosody in reading and language outcomes across all ages.

While traditional measures of phonological skill do not typically include an isolated assessment of prosodic skill, we can gain insight into the genetic underpinnings of prosody, which is a facet of linguistic phonology (i.e., rules governing how units of sound in speech go together in a language),[22] from studies of phonology. To our knowledge, there are no heritability studies focused purely on prosody perception. However, given the shared reliance of both prosody and phonological processing on underlying mechanisms such as auditory discrimination and working memory, heritability estimates for phonological awareness, which range from 0.46 to 0.64,[23,24] suggest that a significant heritable component likely contributes to prosodic skills. We use these estimates as the closest available proxy for the heritability of general speech rhythm perception. Genomic studies of prosodic skills, including speech rhythm perception, may link to reading and other speech-language traits that have been more robustly genetically characterized. Further, uncovering a shared genetic architecture of prosody, speech, language, reading, and/or rhythm could inform education solutions for improved reading and literacy outcomes.

The emerging body of work on the genetic underpinnings of prosody may also have implications for the evolutionary history of language traits. It has been theorized that early communication through prosody-rich proto-language sounds facilitated the survival of groups, and ultimately led to the emergence of even more complex organized patterns (i.e., syntax[25]) in humans. This gradual, pre-adaptive process could have leveraged existing genetic architecture, conserving genetic variation associated with auditory-vocal processing (i.e., the ability to make or perceive these sounds) due to survival benefits (see Kotz et al.[26] for a review of evolution of rhythm processing). Vocal communication in species capable of vocal learning (i.e., species that can modify their own vocalizations as a result of experience with those of other individuals[27–29]) displays a number of features similar to human prosody. The connection between songbirds and humans in particular is noteworthy in that both have the capacity to learn and communicate using complex patterns of sounds ("Revised Vocal Learning Hypothesis"[30,31]). These behavioral similarities are further complemented through similar neurobiological structure and function supporting songbird vocal learning and human communication abilities, including at the genetic level.[32–34] Specifically, the comparative analysis conducted by Mol et al.[35] highlights significant similarities between the suprasegmental organization of human prosody and songbird song. They demonstrate that they both share a hierarchical structure, where fundamental acoustic units are organized into larger, phrase-like groups (e.g., syllables are grouped into motifs, and words are organized into phonological phrases). Importantly, the temporal and melodic characteristics of these structures are influenced by context-dependent acoustic variations that convey essential social and emotional information. Furthermore, both human infants and juvenile songbirds depend significantly on these prosody-like acoustic cues to segment and learn complex vocal signals during their respective developmental stages.

In addition to human language and communication analogs to songbird vocal learning, there is also evidence that vocal learning and human rhythm processing (e.g., beat synchronization) share some degree of common genetic substrates. Given that prosody perception as defined here (in terms of word-level stress patterns) is a fundamentally rhythmic element of spoken language processing, the current work extends these comparative analyses between humans and songbirds (the largest evolutionary radiation of vocal learners) to the domain of prosody perception. Building upon prior evidence, particularly considering evolutionary adaptation and conservation of shared genetic signals, we hypothesize some overlap in the genetic substrates of prosody and songbird vocal learning.

Indeed, underdeveloped sensitivity to speech rhythm perception may be a key component of reading disorders, given that on average both children and adults with dyslexia perform less well on tasks that involve manipulating stress patterns of words than those without dyslexia.[36–38] Impaired rhythm processing, across both speech rhythm and musical rhythm domains, potentially involves less efficient neural subprocesses such as precise auditory timing, synchronization of neural oscillatory activity, and sensorimotor coupling. These subprocesses are hypothesized to play a downstream role in reading and other language-related impairments.[39,40] Behaviorally, individual differences in speech rhythm perception tasks have been explained in part by variance in musical rhythm perception abilities[41–43] pointing to domain-general rhythm processes. Interestingly, recent theories further posit widespread shared genetic influences between musical rhythm and human speech-language traits including prosody perception.[1] A recently published large genetic study of musical rhythm (specifically, the self-reported ability to clap in time with a musical beat, i.e., "beat synchronization") revealed 69 genome-wide significant loci, and downstream biological discovery of musical rhythm abilities in humans.[44] Musical rhythm traits are directly biologically relevant to language abilities as

evidenced by behavioral[1,45] and genetic findings.[7,9] Further, musical rhythm abilities are related specifically to prosody perception.[1,41–43] This recent GWAS of musical rhythm abilities motivates further genetic investigations of prosody in the current work.

Despite its importance for reading and language development and disorders and educational outcomes, the biology of prosody is sparsely understood. In order to gain insights into its biology, and test theories about biological and evolutionary connections between musical rhythm, speech rhythm, and language/reading abilities, we first need to systematically characterize the genetics of prosody perception. Characterizing the genetics of prosody, and consequently understanding the biological associations between prosody, reading, and other rhythm and language-related traits may offer valuable insights into how prosody influences reading outcomes, as well as other speech-language development outcomes. The current study investigates the genetic architecture of speech rhythm perception using population-based genome-wide approaches. We performed a GWAS of a validated speech rhythm perception phenotype in n = 1,501 individuals of European genetic ancestry to identify genomic loci associated with prosody perception scores. To begin to explore the potentially shared evolutionary history between songbird and human communication, we performed gene set enrichment analysis using songbird vocal learning gene sets. Last, to evaluate the genetic relationships between prosody and other phenotypically relevant traits, we estimated if polygenic scores (PGSs) for (1) word reading, (2) beat synchronization, and (3) voice pitch variability in reading (which correlates phenotypically with verbal fluency and reading measures),[3] predict prosody perception scores in an independent sample. Together, this research is an important step toward understanding shared etiological relationships between prosody and other speech, language, and literacy outcomes.

Subjects and Methods

Phenotyping

Test for Prosody via Syllable Emphasis (“TOPsy”) is a 28-item word-level stress identification task that requires participants to identify which syllable carries the primary emphasis or stress in a series of multi-syllabic spoken words.[41] TOPsy is a highly reliable and validated task that was specifically designed to improve phenotyping in the context of large-scale genomics research. TOPsy has several features that address common trade-offs between characterizing phenotypes richly, and achieving large sample sizes. For instance, TOPsy allows for remote testing and automatic scoring (not possible for existing prosody perception tests), while improving reliability compared with other existing prosody tests (Cronbach’s $\alpha = 0.92$). Further, TOPsy counterbalances syllable length and syllable stress position across items tested, a feature not previously incorporated into other prosody perception tests.[19,46] The 28 test items were finalized through exploratory factor analysis, and the test takes 10 min to complete remotely, making it ideal for human genomics research. Development and validation of TOPsy were previously reported elsewhere (see Nayak et al.[41]).

Study design

The study population was a subset of those who participated in the *Vanderbilt Online Musicality Study*,[47] where participants completed various internet-based tasks, including TOPsy, and submitted genetic samples. A total of 1,698 participants (n = 1,501 with European genetic ancestry) completed the TOPsy task. Due to substantial skew in the data, data from the TOPsy task were inverse-rank transformed. Specifically, a linear model was fit adjusting for age, sex, and first five ancestry principal components (PCs); and residuals of this model were inverse-rank transformed, and used as the new speech rhythm perception phenotype. All analyses were performed focusing on those with European genetic ancestry, and using transformed speech rhythm perception (TOPsy) scores, unless otherwise specified (i.e., in PGS analyses). All participants provided informed consent prior to participation, and study procedures were approved by Vanderbilt's Institutional Review Board.

Genotyping, quality control procedures, and imputation

Participants were genotyped using the Illumina Expanded Multi-Ethnic Genotyping Array ($MEGA^{EX}$) at VANTAGE, Vanderbilt University Medical Center’s core facility. Genetic data quality was assessed and missing variants were imputed as part of the Vanderbilt Online Musicality Study (see supplement in Gustavson et al.[47] for more details).

Briefly, sample and variant filtering was performed in PLINK v.1.90[48] using the following parameters: minor allele frequency <0.01, variant missingness > 0.10, and sample missingness > 0.15. Samples that failed the heterozygosity (F > 0.2 or < -0.2) and sex checks (mismatch in imputed and expected biological sex) were removed. PC analysis was performed on the maximum unrelated set using PC-Air to determine genetic ancestry.[49] Kinship was then estimated with PC-Relate.[50] After, the data were filtered again to remove samples with missingness > 0.05 and Hardy-Weinberg equilibrium $p < 1 \times 10^{-8}$. Data were then phased using Eagle v.2.4[51] and then imputed to TOPMed Imputation Server[52] using Minimac4.[53] Post-imputation quality control filtering consisted of removing variants with a minor allele frequency < 0.01 and imputation quality $R^2 < 0.70$ using BCFtools.[54] Using this cleaned dataset from the Vanderbilt Online Musicality Study,[47] we identified the maximum unrelated set (up to third degree).[49]

Genome-wide association analysis

Using the transformed speech rhythm perception phenotype (see study design), ∼7 million imputed variants were analyzed for their association with prosody perception scores in PLINK2[48] using a linear regression model through the –glm command. Model covariates included age, sex, and the first five PCs explaining population substructure variation (based on the number of ancestry PCs that were correlated with the phenotype). Since GWAS model covariates were the same ones included during the phenotype transformation step (see study design) based on guidance in Sofer et al.,[55] we also conducted the GWAS without the covariates to report results without the two-stage procedure (see supplemental information). Sentinel variants were defined as the most

significant variant found within a ±1 MB window, and surpassed a minor allele count of 30.

Annotation

We annotated genome-wide significant sentinel variants ($p < 5.00 \times 10^{-8}$) and suggestive significant signals ($p < 5.00 \times 10^{-6}$) using the Open Targets Genetics Variant-to-Gene (V2G) pipeline, which aggregates evidence from chromatin interactions, molecular quantitative trait loci, *in silico* functional predictions, and distance between the variant with the gene transcription start site.[56,57] All reported positional coordinates (chromosome and base pair locations) refer to human genome reference build 37.

Gene-based GWAS

Prior to performing our gene-based association analysis, we first annotated our autosomal SNPs to protein coding genes using the –annot command in MAGMA (v.1.09)[58] and specifying a SNP annotation window of +35/−10kb.[59] Next, we performed gene-based association analysis using MAGMA (v.1.09)[58] on our individual-level genetic data and speech rhythm perception scores, controlling for age, sex, and first five PCs. Genome-wide significance was determined using a p value threshold < 2.71×10^{-6}, a Bonferroni correction for the 18,441 genes tested.

Gene set enrichment analysis of birdsong gene sets

We collated seven birdsong gene sets that were generated from microarray studies of zebra finch to find genes differentially expressed in association with singing behaviors.[60] Set 3: singing vs. silence (Area X); set 5: singing vs. silence (robust nucleus of the arcopallium "RA"); set 6: singing vs. silence (the lateral magnocellular nucleus of the anterior nidopallium "LMAN"); set 7: singing vs. silence (Area X: controlling for differential expression in genes in the ventral striato-palladium "Ctrl VSP," a non-singing-specific network in songbird brains); set 8: number of motifs sung (Area X: Ctrl VSP); set 10: listening/playback (two auditory areas, caudal medial nidopallium and the L2a portion of Field L, in males that heard song vs. silence). See Gordon et al.[60] for details on gene set creation and access to curated gene sets. We then tested the enrichment of these gene sets in human speech rhythm perception using MAGMA (v.1.09).[58] The birdsong gene sets tested were limited to sets containing more than 20 genes. Since Area X is a known ortholog of the basal ganglia,[61,62] we created an "Area X overlap gene set," which was the intersection of the 477 overlapping genes across all Area X birdsong gene sets (i.e., set 3: singing vs. silence [Area X]; set 7: singing vs. silence [Area X Ctrl VSP]; and set 8: number of motifs sung [Area X Ctrl VSP]). Gene set analysis significance was determined to be $p < .0125$, a Bonferroni correction for the four distinct brain regions of these gene sets (Area X: sets 3, 7, 8, and overlap; LMAN: set 6; RA: set 5; auditory areas: set 10). For gene set enrichment results with unadjusted p values < .05, we performed conditional gene set analysis conditioning on average gene expression[63] in the brain[64] (see supplemental information).

PGS construction and testing

PGS models were trained using prior GWAS results for our three traits of interest: (1) word reading[2] ($n = 27{,}180$), (2) beat synchronization[44] ($n = 606{,}825$), and (3) voice pitch variability in reading[3] ($n = 12{,}901$) using PRS-CS[65]. PRS-CS is a Bayesian regression method that places a continuous shrinkage prior on individual SNP weights for LD and variant significance. PGS models were trained using European genetic ancestry GWAS summary statistics. A European LD reference constructed from 1000 Genomes Project phase 3 was used for all analyses. Default auto-phi parameters were used to prevent model overfitting. The speech rhythm perception testing set ($n = 1{,}698$ individuals from all genetic ancestries, "multi-ancestry sample") was scored using PLINK v.1.9.[48]

Similar to methods used in Gustavson et al.,[47] PGSs were Z scored within each of the five genetic ancestry groups to reflect standardized associations between our trait of interest and speech rhythm perception scores. To determine if the genetic predisposition of our trait of interest predicted speech rhythm perception scores, we performed regression models where the primary outcome was speech rhythm perception scores regressed on the within-ancestry Z scored PGS scores, controlling for age, sex, and the first five PCs. All measures in the model were standardized. Our analyses focused on using the full multi-ancestry sample ($n = 1{,}698$). We also present analyses restricted to only individuals of European genetic ancestry ($n = 1{,}501$) in the supplemental information. Table 1 shows the sample sizes and age distributions for genetic ancestry groups.

Results

Speech rhythm perception TOPsy scores

To avoid ceiling effects, speech rhythm perception scores were transformed. A linear model for TOPsy scores was fit adjusting for age, sex, and first five ancestry PCs, and the inverse-rank transformed residuals of the model was used as the speech rhythm perception phenotype (see study design, Table 1). TOPsy scores were available for $n = 1{,}729$ people who also had clean genotyped data. Thirteen individuals were removed due admixture, 13 were removed due to relatedness (see subjects and methods), and 5 were dropped from models due to missing covariate information (i.e., age or sex). Hence, our total sample size was $n = 1{,}698$. Further phenotypic analyses of TOPsy scores in a larger overall sample which includes individuals without genotyping ($n = 2{,}508$), have been previously detailed.[41]

Genome-wide association analysis

We performed a GWAS of speech rhythm perception, where our phenotype was based on performance on the TOPsy task.[41] GWAS was carried out in 1,501 unrelated individuals of European genetic ancestry with ∼7 million variants tested (Table 1). The genomic inflation factor, λ, was 1.0065 (Figure 1). Although no variants surpassed genome-wide significance ($p < 5.00 \times 10^{-8}$), 14 loci reached suggestive significance ($p < 5.00 \times 10^{-6}$, Table 2). In supplementary analyses, we mapped these 14 signals to genes using the Open Targets V2G pipeline[53,54] and queried these genes within the GWAS Catalog.[66] We found that our speech rhythm perception-associated genes were previously associated with the following broad trait categories: Cardiac/Circulatory, Obesity/Endocrine/Metabolic, Metabolites, Neurological, and Mental Disorders (see supplemental information). Since the GWAS model

Table 1. Demographic information and overview of speech rhythm perception performance

Genetic ancestry	*n* (% female)	Age (SD)	Raw % TOPsy scores (SD)	Median raw % TOPsy scores (range)
All	1,698 (74.17%)	45.11 (16.26)	76.75 (22.85)	84.38 (16.25, 100)
African	90 (90.00%)	43.42 (14.42)		
Hispanic/Latino	33 (69.70%)	34.09 (13.51)		
East Asian	49 (77.55%)	34.92 (12.72)		
South Asian	25 (52.00%)	31.20 (9.07)		
European	1,501 (73.55%)	46.02 (16.32)	77.68 (22.57)	86.25 (16.25, 100)

Genetic ancestry was derived by PC analysis. Due to ceiling effects, a linear model for TOPsy scores was fit adjusting for age, sex, and first five ancestry PCs. The residuals of the model were inverse ranked transformed and used as the speech rhythm perception phenotype (see subjects and methods).

covariates were included during the phenotype transformation step[55] (see study design), we further performed the GWAS without covariates (see supplemental information, Table S2; Figure S2).

Gene-based GWAS

To better prioritize genes associated with speech rhythm perception, we conducted a gene-based GWAS using MAGMA (v.1.09).[58] Although our gene-based GWAS did not yield any genes surpassing Bonferroni significance ($p < 2.71 \times 10^{-6}$), top associated genes consisted of *TTLL1*, *GP2*, *C8B*, and *HEPACAM2*, $p < 1.42 \times 10^{-4}$ (Figure 2).

Gene set analysis

To explore the potential for evolutionary convergence between speech rhythm perception and other vocal learning behaviors, we performed gene set enrichment analysis using sets of genes that were differentially expressed in association with singing behaviors in zebra finches (see Gordon et al.[60] for more details). Human speech rhythm perception was enriched for singing vs. silence in Area X and the Area X overlap gene set, $p < .05$ (Figure 3). No gene sets surpassed Bonferroni correction ($p < .0125$, a correction for the four brain regions spanning the birdsong gene sets (**Area X**: sets 3, 7, 8, and overlap; **LMAN**: set 6; **RA**: set 5; **auditory areas**: set 10). See Table 3 for full gene set enrichment analysis results. Conditional gene set analysis[63] results controlling for average brain expression[64] as a gene property can be found in Table S3.

PGS analysis of word reading

Speech rhythm perception and reading abilities are robustly phenotypically correlated in both children[16,17,67–69] and adults,[19,46] with prosody being recently highlighted as a missing factor in current models of reading and literacy development.[19] To examine if there are relevant biological signals connecting speech rhythm perception and reading, we sought to determine if genetic predispositions for word reading abilities explain variability in speech rhythm perception scores. Word reading PGSs were derived from word reading summary statistics from the GenLang consortium.[2] Genetic predispositions for word reading were positively correlated with speech rhythm perception phenotypes ($\beta = 0.109$; $p = 2.23 \times 10^{-5}$; 95% CI, 0.060, 0.160;

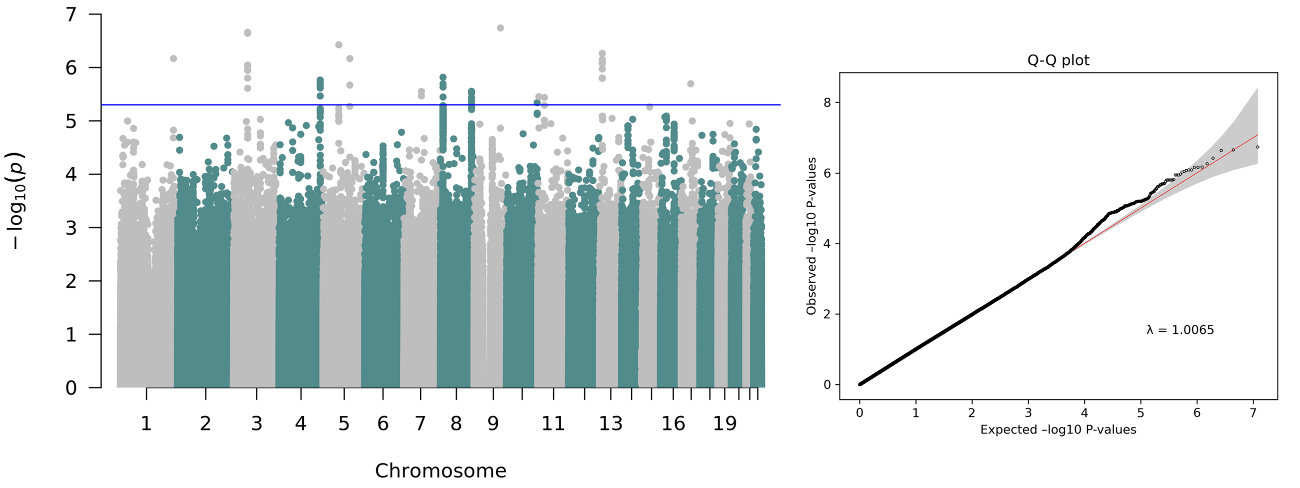

Figure 1. Manhattan plot and Q-Q plot for genome-wide association analysis of speech rhythm perception
Genome-wide association analysis included 1,501 individuals of European genetic ancestry and 6,778,702 variants. Results showed 14 loci of interest at a suggestive significance p value threshold $< 5.00 \times 10^{-6}$ (indicated by the blue line). Q-Q plot x axis represents expected $\log_{10} p$ and the y axis represents observed $-\log_{10} p$.

Table 2. Suggestive significant signals associated with speech rhythm perception

rsid	CHR	POS_b37	BETA	EA	NEA	EAF	SE	Functional gene(s)	*p* value
rs146064482	9	114369425	−0.719	T	G	0.018	0.137	*GNG10*	1.81E−07
rs56702966	3	60958999	0.301	C	T	0.11	0.058	*FHIT*	2.19E−07
rs6886492	5	68425699	−0.235	G	A	0.188	0.046	*SLC30A5*	3.75E−07
rs3000634	13	31269282	0.314	G	A	0.095	0.062	*ALOX5AP*	5.41E−07
rs80288146	1	231061747	0.597	T	C	0.024	0.12	*TTC13*	6.79E−07
rs67704630	5	116506760	0.401	C	T	0.054	0.08	NA	6.81E−07
rs11250130	8	11214455	0.178	A	G	0.473	0.037	*FAM167A*	1.52E−06
rs6837755	4	178680187	−0.183	A	G	0.325	0.038	*AGA*	1.71E−06
rs138312553	17	41023343	0.593	G	A	0.021	0.124	*AOC3*	2.01E−06
rs34483201	8	135010378	−0.187	T	C	0.3	0.04	*ST3GAL1*	2.79E−06
rs139396308	7	76384707	−0.746	A	G	0.013	0.159	*POMZP3*	2.81E−06
rs6578580	11	5214116	−0.174	C	T	0.424	0.037	*OR51V1*	3.51E−06
rs192407100	11	29370196	−0.536	T	C	0.026	0.115	*BDNF*	3.64E−06
rs12774548	10	132984505	−0.26	T	G	0.117	0.057	*TCERG1L*	4.57E−06

All signals presented are identified as the most significant variant found within a ±1 MB window ($p < 5.00 \times 10^{-6}$). rsid, SNP; CHR, chromosome; POS_b37, position in hg37; BETA, the effect; EA, effect allele; NEA, non-effect allele; EAF, effect allele frequency; SE, standard error; functional gene(s), most likely implicated functional gene that was mapped using Open Targets Genetics; *p* value, association *p* value.

$n = 1{,}698$; Figure 4A), after controlling for age, sex, and the first five ancestry PCs. These findings were replicated when tested only in individuals of European genetic ancestry (see supplemental information, Table S4; Figure S3A). Further comparison of word reading and speech rhythm perception GWAS results through concordance analysis can be found in Table S5.

PGS analysis of beat synchronization

Musical rhythm and speech rhythm perception are also known to be phenotypically correlated; with rhythm and language traits (including prosody perception) being theorized to have underlying shared genetic architecture.[1] To observe if a shared genetic etiology connects beat synchronization and speech rhythm perception, we tested if the genetic predisposition of beat synchronization predicts speech rhythm perception scores. Beat synchronization scores were based on a recent GWAS.[25] We observed that the genetic predisposition of beat synchronization explains speech rhythm perception skills ($\beta = 0.138$; $p = 1.11 \times 10^{-8}$; 95% CI, 0.09, 0.18; $n = 1{,}698$; Figure 4B), after controlling for age, sex and, the first five PCs. These findings were replicated when testing in individuals of European genetic ancestry (see supplemental information, Table S4; Figure S3B). Further comparison of beat synchronization and speech rhythm perception GWAS results through concordance analysis can be found in Table S5.

PGS analysis of voice pitch variability

Recent efforts have aimed to uncover the genetic architecture of voice pitch and vowel acoustics.[3] Voice pitch variability in reading is phenotypically correlated with higher performance on verbal fluency tasks and reduced reading difficulties, and genetically correlated with education. To

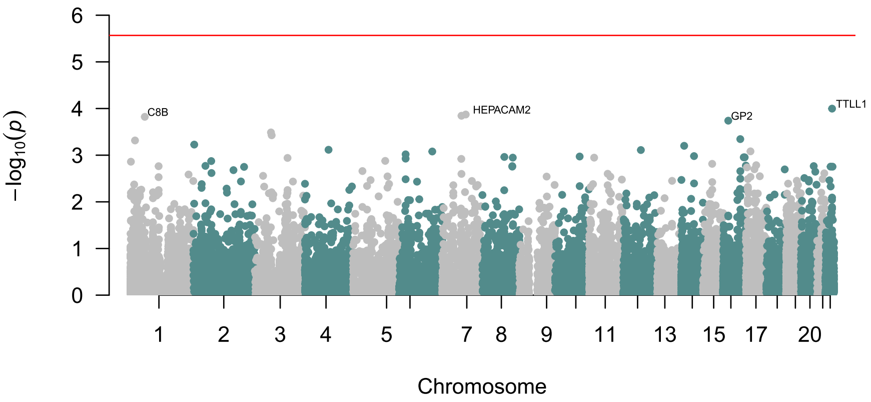

Figure 2. Gene-based genome-wide association analysis of speech rhythm perception

Gene-based genome-wide association analysis included 1,501 individuals of European genetic ancestry and 18,441 genes. The red line indicates Bonferroni significance, $p < 2.71 \times 10^{-6}$, 0.05/18,441 genes tested.

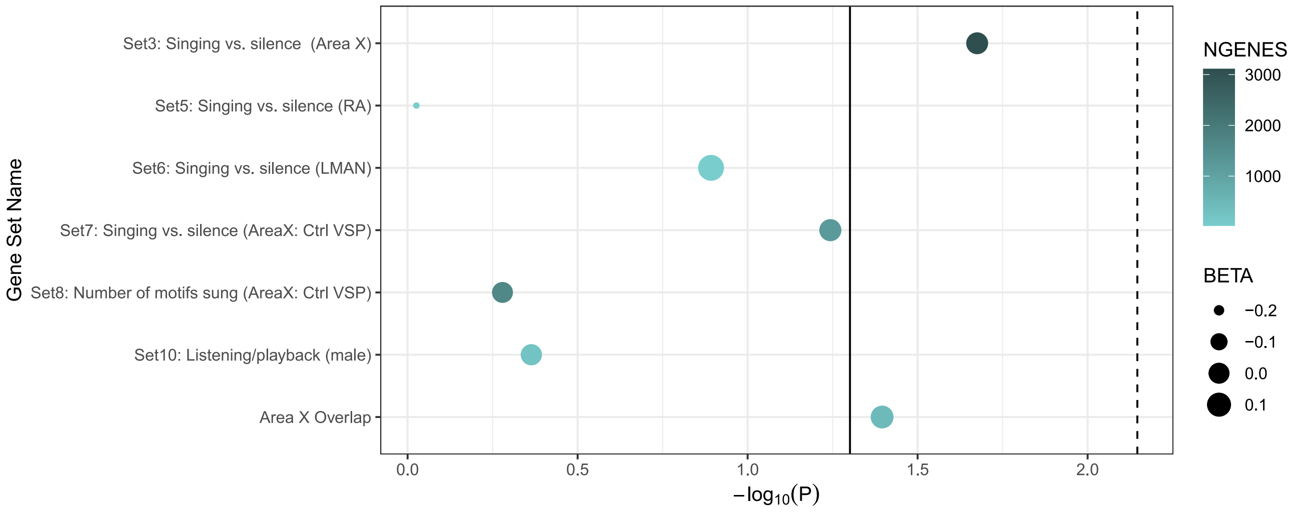

Figure 3. Birdsong gene set enrichment analysis of speech rhythm perception
Gene sets were obtained from Gordon et al. 2021[60]. The solid line represents nominal significance, $p < .05$. The dotted line represents the Bonferroni gene set analysis significance, $p < .0125$, a Bonferroni correction for the four distinct brain regions sampled in the birdsong gene sets. Area X, a known ortholog of the human basal ganglia; Ctrl ventral striato-pallidum ("Ctrl VSP"), controlling for the differential expression of genes in a brain region involved in non-vocal motor function); LMAN, the lateral magnocellular nucleus of the anterior nidopallium; RA, robust nucleus of the arcopallium.

explore shared genetic signals connecting speech rhythm perception and voice pitch variability in speech production, both aspects of prosody perception and production, we tested if the genetic predisposition of voice pitch variability predicts speech rhythm perception scores. Voice pitch variability scores were obtained from a recent GWAS on voice pitch and vowel acoustics.[3] We found that the genetic predisposition of voice pitch variability predicts speech rhythm perception skills ($\beta = 0.062$; $p = 1.08 \times 10^{-2}$; 95% CI, −0.428, 0.552; $n = 1{,}698$; Figure 4C), after controlling for age, sex, and the first five PCs. However, this finding did not replicate when testing only in individuals of European genetic ancestry (see supplemental information, Table S4; Figure S3C).

Discussion

The current study performs a genome-wide investigation of speech rhythm perception (an aspect of speech prosody perception), which is an important yet often overlooked aspect of speech and language abilities. Although no loci surpassed genome-wide significance ($p < 5.00 \times 10^{-8}$), GWAS results revealed 14 signals reaching suggestive significance ($p < 5.00 \times 10^{-6}$). Results of polygenic score analyses also suggest a common genetic architecture connecting speech rhythm perception and language, reading, and musical rhythm (i.e., beat synchronization) traits. Further, we showed that human prosody perception shares genetic signatures with key vocal learning brain regions in songbirds, consistent with theories of evolutionary convergence of communication mechanisms across species.

Since our sample size was underpowered (Figure S4) and did not reveal any genome-wide signals, we further explored the 14 signals that reached suggestive significance to begin to map the biology of prosody and point to directions for future research. Mapping these 14 loci to genes and querying them in the GWAS Catalog[66] revealed biological relevance related to the following broad trait categories: Cardiac/Circulatory,

Table 3. Gene set analysis for speech rhythm perception

Gene set	NGENES	BETA	SD	SE	*p* value
Set 3: Singing vs. silence (Area X)	3112	0.028	0.011	0.014	0.021
Area X overlap	462	0.058	0.009	0.033	0.040
Set 7: Singing vs. silence (Area X: Ctrl VSP)	1204	0.034	0.008	0.021	0.057
Set 6: Singing vs. silence (LMAN)	24	0.170	0.006	0.149	0.128
Set 10: Listening/playback (male)	261	0.007	0.001	0.042	0.433
Set 8: Number of motifs sung (Area X: Ctrl VSP)	1670	−0.001	0.000	0.019	0.526
Set 5: Singing vs. silence (RA)	26	−0.216	−0.008	0.137	0.942

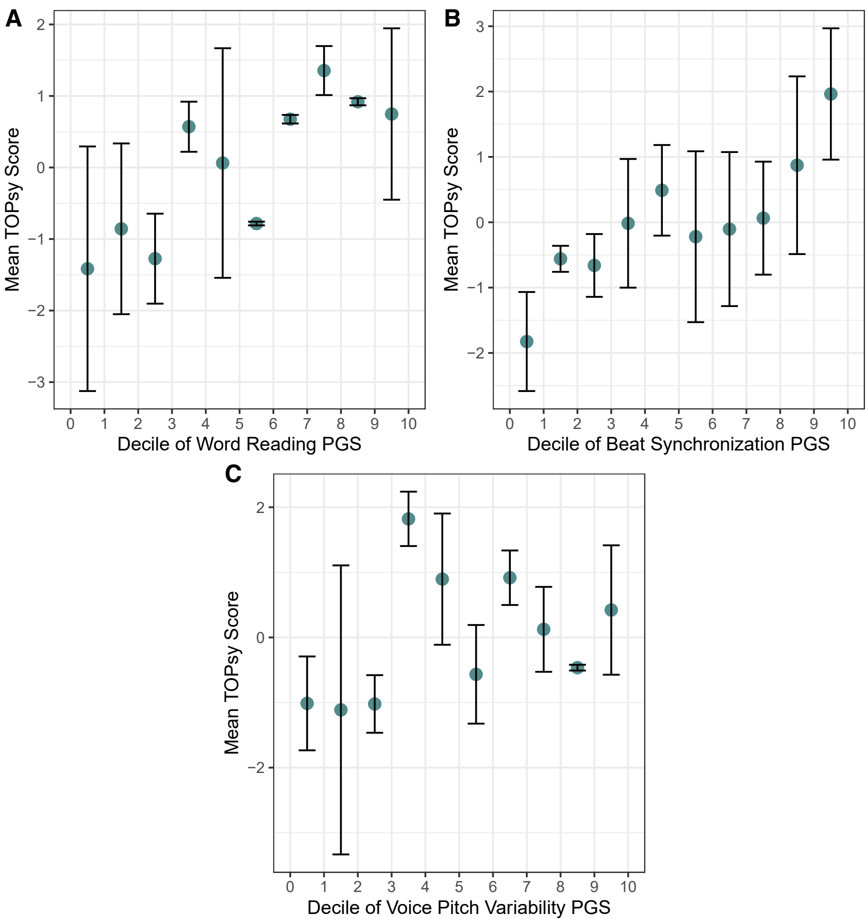

Figure 4. Polygenic associations between speech rhythm perception and other musicality and language traits
Decile plots showing polygenic scores of (A) word reading, (B) beat synchronization, and (C) voice pitch variability on the x axis, and behavioral speech rhythm perception scores on the y axis, in $n = 1,698$. Polygenic scores were derived from GWAS results reported in Eising et al.,[2] Niarchou et al.,[44] and Gisladottir et al.,[3] respectively. Polygenic scores and behavioral scores with standard error bars are visualized for $n = 1,698$ individuals from the full sample.

Obesity/Endocrine/Metabolic, Metabolites, Neurological, and Mental Disorders. These findings are particularly interesting given that impairments in prosody have been observed in various mental disorders and neurological conditions, such as autism spectrum disorder,[70] depression,[71] right-brain damage,[72] and Alzheimer's disease.[73] Although most prior studies investigating prosody and mental health or neurological disorders primarily focused on prosody *production*, our findings about prosody *perception* converge with these previous investigations, potentially highlighting its underestimated relevance for mental and neurological disorders. Prosody perception is therefore an area ripe for future study in the context of mental health, psychiatric, and neurological domains, among others.

Individual-level cross-trait PGS analyses offer additional validation of, and confidence in, the speech rhythm perception phenotype (i.e., TOPsy scores). As predicted, musical rhythm and reading traits—both known to be phenotypically correlated with prosody perception—also exhibited relevant biological connections with it. Specifically, genetic predispositions for word reading abilities predicted speech rhythm perception scores in an independent sample. These results reveal biological evidence consistent with long-standing findings about the importance of prosody perception in reading outcomes across the lifespan.[16,17,19,46,67–69,74,75] Similarly, musical rhythm skills are a known behavioral and neural correlate of speech rhythm abilities[39,42,43,76] and were also genetically associated in PGS analyses, consistent with the Musical Abilities, Pleiotropy, Language, and Environment (MAPLE) framework,[1] which posits that shared genetic influences partially drive the widespread associations seen between several musicality and speech-language traits. Taken together, these findings reiterate the relevance of genetically influenced neural endophenotypes that may underlie individual differences in these intercorrelated traits (i.e., prosody, musical rhythm, reading).

Our gene-based GWAS revealed that *TTLL1* and *GP2* genes were in the top 5 most highly associated with speech rhythm perception, although no genes surpassed statistical thresholds for genome-wide significance. *TTLL1* is involved in microtubule organization,[77] which in turn is important for cellular morphology and function, including those involved in neurodevelopmental, degeneration, and regeneration processes.[78] Additionally, *GP2* has been associated with sleep quality[79] and endocrine-related traits.[80,81] *GP2*'s association with sleep quality is particularly interesting given that the genetic architecture of beat synchronization, capturing an aspect of musical rhythm perception, was correlated with other biological-rhythm traits, including circadian chronotype and insomnia.[44] These findings begin to shed light on the potential biological mechanisms involved in speech rhythm perception, and should be validated in future well-powered samples.

Cross-species comparisons further shed light on the potential evolutionary convergence of human speech rhythm perception and vocal learning traits in other taxa. Genes associated with our prosody perception phenotype were enriched for genes expressed in Area X of the songbird brain during birdsong. Area X is a known

ortholog for the human basal ganglia,[61,62] which has been previously studied for its relevance to musical rhythm abilities and neural processing.[82–86] Relatedly, previous studies have shown that genes associated with human musical beat synchronization (i.e., musical rhythm abilities) are enriched for genes expressed during birdsong,[60] complementing genetic and neural similarities in the cortico-basal-ganglia circuitry across species.[33] These results demonstrate fundamental connections between prosody perception in humans and other vocal learning species and are consistent with theories of language evolution. Current theories posit that early prosody-rich protolanguage sounds facilitated survival and led to the emergence of complex language (i.e., syntax).[25] Further, facets of human speech and language abilities are thought to have convergently evolved from pre-adaptations in vocal learning species more broadly.[30,31]

Some limitations of the study include small sample size and overrepresentation of individuals of European genetic ancestry (~88% of our sample). Notably, our sample size lacked the sufficient power (Figure S4) to identify speech rhythm perception-associated variants (especially since common genetic variants have small to moderate effects), and further reliably estimate SNP-based heritability. There are also limitations to GWAS more generally, including lack of power to detect modest and low effects at rare variants, challenges related to the quality and specificity of phenotypes, inability to distinguish protective from deleterious effects, and frequent overrepresentation of individuals of European genetic ancestry. Increased sample size and diversity can be achieved by deploying the TOPsy task and/or including questions pertaining to speech and rhythm within large biobank studies. Indeed, the TOPsy task's design enables large-scale rapid phenotyping and can facilitate future genomics and epidemiological efforts, as well as more targeted investigations of prosody perception in psychiatric and neurological disorder contexts. Further, since these genomic findings were captured from the same participants as those involved in the validation of the phenotyping tool TOPsy,[41] future research should replicate phenotypic and genomic efforts related to prosody perception to ensure rigor and external validations. Given the paucity of genetics studies of speech rhythm perception, these initial results are promising and should serve as potential avenues for future validation and investigation of other language and reading traits. Additionally, it is important to highlight that, although prosody is typically phenotypically linked to phonological processing skills, the manifestation of prosodic skills is a multi-dimensional phenomenon encompassing a range of acoustic features, communicative functions, and cognitive processes. The complexity of prosody can potentially explain why our analyses linking speech rhythm perception to PGSs of another prosody phenotype (i.e., voice pitch variability while reading) were inconclusive. Indeed, both of these constructs were measuring different components of prosody, emphasizing the need for diverse and nuanced phenotyping and well-powered study designs.

This study represents a foundational step in understanding the biology relating to individual differences in prosody perception. Our findings lay the groundwork for further exploration into the genetic architecture of speech-language traits; genetic epidemiology linking speech and language function to health more broadly; and educationally relevant outcomes such as reading development and adult literacy. These future directions could improve precision efforts in both communication sciences and disorders and educational settings.

Data and code availability

- For the subset of individuals with speech rhythm perception scores, genotype data for the Vanderbilt Online Musicality Study will be made available to qualified investigators in dbGaP.
- Speech rhythm perception summary statistics genome-wide are available publicly through the GWAS Catalog. The accession number for the speech rhythm perception summary statistics is GWAS Catalog: GCST90809464.
- GWAS summary statistics are available publicly for word reading through the GWAS Catalog (GWAS Catalog: GCST90104463) or through the GenLang network website (see web resources).
- GWAS summary statistics for voice pitch variability and beat synchronization are restricted based on data use agreements.

Acknowledgments

We acknowledge feedback from these additional colleagues, who served as members of our project advisory board: Drs. Matthew Leonard, Duane Watson, and Jennifer Zuk. We would like to thank participants from 23andMe and the Vanderbilt Online Musicality Study. This work was supported by funding from the National Science Foundation (NSF 1926794 and NSF 1926736) and by funding from the National Institute on Deafness and Other Communication Disorders (NIDCD), Office of the Director (OD), and National Institute on Drug Abuse (NIDA), of the National Institutes of Health (NIH), under award numbers R01DC016977, R01DC017175, R01DA059804, R21DC021276, R03DC021550, and F31DC022482. Data collection and management via REDCap was made possible through awards from the NCATS/NIH (UL1 TR000445). The content is solely the responsibility of the authors and does not necessarily represent the official views of the funders.

Author contributions

R.L.G. and S.N. oversaw study; R.L.G. designed the study, with conceptual contributions from S.N., N.C., J.E.B., N.J.C, and R.S.G; A.C.S., S.N., and R.L.G. drafted and revised the manuscript, with contributions from J.E.B., L.E.P., H.M.H., N.C., D.E.G., R.S.G., and C.L.M; A.C.S. and S.N. performed analyses, with contributions from P.L.C., T.L.H., and X.L.D; Y.W. and D.E.G. oversaw data

management; P.L.C. performed quality control on the genetic data. All authors critically reviewed the manuscript.

Declaration of interests

The authors declare no competing interests.

Supplemental information

Supplemental information can be found online at https://doi.org/10.1016/j.xhgg.2026.100581.

Web resources

GenLang network, https://www.genlang.org/

GWAS Catalog, https://www.ebi.ac.uk/gwas/

Open Targets Genetics, https://platform.opentargets.org/

Received: March 27, 2025
Accepted: February 9, 2026

References

1. Nayak, S., Coleman, P.L., Ladányi, E., Nitin, R., Gustavson, D.E., Fisher, S.E., Magne, C.L., and Gordon, R.L. (2022). The Musical Abilities, Pleiotropy, Language, and Environment (MAPLE) Framework for Understanding Musicality-Language Links Across the Lifespan. Neurobiol. Lang. *3*, 615–664.
2. Eising, E., Mirza-Schreiber, N., de Zeeuw, E.L., Wang, C.A., Truong, D.T., Allegrini, A.G., Shapland, C.Y., Zhu, G., Wigg, K.G., Gerritse, M.L., et al. (2022). Genome-wide analyses of individual differences in quantitatively assessed reading- and language-related skills in up to 34,000 people. Proc. Natl. Acad. Sci. USA *119*, e2202764119.
3. Gisladottir, R.S., Helgason, A., Halldorsson, B.V., Helgason, H., Borsky, M., Chien, Y.R., Gudnason, J., Gudjonsson, S.A., Moisik, S., Dediu, D., et al. (2023). Sequence variants affecting voice pitch in humans. Sci. Adv. *9*, eabq2969.
4. Doust, C., Fontanillas, P., Eising, E., Gordon, S.D., Wang, Z., Alagöz, G., Molz, B., 23andMe Research Team, Quantitative Trait Working Group of the GenLang Consortium, Pourcain, B.S., et al. (2022). Discovery of 42 genome-wide significant loci associated with dyslexia. Nat. Genet. *54*, 1621–1629.
5. Polikowsky, H.G., Shaw, D.M., Petty, L.E., Chen, H.H., Pruett, D.G., Linklater, J.P., Viljoen, K.Z., Beilby, J.M., Highland, H.M., Levitt, B., et al. (2022). Population-based genetic effects for developmental stuttering. HGG Adv. *3*, 100073.
6. Shaw, D.M., Polikowsky, H.P., Pruett, D.G., Chen, H.H., Petty, L.E., Viljoen, K.Z., Beilby, J.M., Jones, R.M., Kraft, S.J., and Below, J.E. (2021). Phenome risk classification enables phenotypic imputation and gene discovery in developmental stuttering. Am. J. Hum. Genet. *108*, 2271–2283.
7. Polikowsky, H.G., Scartozzi, A.C., Shaw, D.M., Pruett, D.G., Chen, H.H., Petty, L.E., Petty, A.S., Lowther, E.J., Cho, S.H., Yu, Y., et al. (2025). Large-scale genome-wide analyses of stuttering. Nat. Genet. *57*, 1835–1847.
8. de Hoyos, L., Verhoef, E., Okbay, A., Vermeulen, J.R., Figaroa, C., Lense, M., Fisher, S.E., Gordon, R.L., and St Pourcain, B. (2025). Preschool musicality is associated with school-age communication abilities through genes related to rhythmicity. npj Sci. Learn. *10*, 39. https://doi.org/10.1038/s41539-025-00329-y.
9. Alagöz, G. (2024). The shared genetic architecture and evolution of human language and musical rhythm. Nat. Hum. Behav. *9*, 376–390.
10. Mekki, Y., Guillemot, V., Lemaître, H., Carrión-Castillo, A., Forkel, S., Frouin, V., and Philippe, C. (2022). The genetic architecture of language functional connectivity. Neuroimage *249*, 118795.
11. Jusczyk, P.W., Cutler, A., and Redanz, N.J. (1993). Infants' Preference for the Predominant Stress Patterns of English Words. Child Dev. *64*, 675–687.
12. Redford, M.A., and Oh, G.E. (2016). Children's abstraction and generalization of English lexical stress patterns. J. Child Lang. *43*, 338–365.
13. Snedeker, J., and Trueswell, J. (2003). Using prosody to avoid ambiguity: Effects of speaker awareness and referential context. J. Mem. Lang. *48*, 103–130.
14. Hupp, J.M., and Jungers, M.K. (2013). Beyond words: Comprehension and production of pragmatic prosody in adults and children. J. Exp. Child Psychol. *115*, 536–551.
15. Adams, M. (1990). Beginning to Read: Thinking and Learning about Print (Cambridge: The MIT Press).
16. Holliman, A. (2016). Suprasegmental phonology and early reading development: Examining the relative contribution of sensitivity to stress, intonation and timing. In Linguistic Rhythm and Literacy (John Benjamins Publishing Company), pp. 25–50.
17. Holliman, A.J., Wood, C., and Sheehy, K. (2010). Sensitivity to speech rhythm explains individual differences in reading ability independently of phonological awareness. Br. J. Dev. Psychol. *26*, 357–367.
18. Clin, E., Wade-Woolley, L., and Heggie, L. (2009). Prosodic sensitivity and morphological awareness in children's reading. J. Exp. Child Psychol. *104*, 197–213.
19. Heggie, L., and Wade-Woolley, L. (2018). Prosodic awareness and punctuation ability in adult readers. Read. Psychol. *39*, 188–215.
20. Fodor, J.D. (1998). Learning to parse? J. Psycholinguist. Res. *27*, 285–319.
21. Breen, M. (2014). Empirical Investigations of the Role of Implicit Prosody in Sentence Processing. Lang. Linguist. Compass *8*, 37–50.
22. Ladd, D.R. (2014). Defining prosody. In Simultaneous Structure in Phonology (Oxford University Press). https://doi.org/10.1093/acprof:oso/9780199670970.003.0003.
23. Andreola, C., Mascheretti, S., Belotti, R., Ogliari, A., Marino, C., Battaglia, M., and Scaini, S. (2021). The heritability of reading and reading-related neurocognitive components: A multi-level meta-analysis. Neurosci. Biobehav. Rev. *121*, 175–200.
24. Samuelsson, S., Olson, R., Wadsworth, S., Corley, R., DeFries, J.C., Willcutt, E., Hulslander, J., and Byrne, B. (2006). Genetic and environmental influences on prereading skills and early reading and spelling development in the United States, Australia, and Scandinavia. Read. Writ. *20*, 51–75.
25. Fitch, W.T. (2017). Empirical approaches to the study of language evolution. Psychon. Bull. Rev. *24*, 3–33.
26. Kotz, S.A., Ravignani, A., and Fitch, W.T. (2018). The Evolution of Rhythm Processing. Trends Cogn. Sci. *22*, 896–910.

27. Janik, V.M., and Slater, P.J. (2000). The different roles of social learning in vocal communication. Anim. Behav. *60*, 1–11.
28. Searcy, W.A., Soha, J., Peters, S., and Nowicki, S. (2021). Variation in vocal production learning across songbirds. Philos. Trans. R. Soc. Lond. B Biol. Sci. *376*, 20200257.
29. Vernes, S.C., Janik, V.M., Fitch, W.T., and Slater, P.J.B. (2021). Vocal learning in animals and humans. Philos. Trans. R. Soc. Lond. B Biol. Sci. *376*, 20200234.
30. Patel, A.D. (2006). Musical Rhythm, Linguistic Rhythm, and Human Evolution. Music Percept. *24*, 99–104.
31. Patel, A.D. (2021). Vocal learning as a preadaptation for the evolution of human beat perception and synchronization. Philos. Trans. R. Soc. Lond. B Biol. Sci. *376*, 20200326.
32. Jarvis, E.D. (2019). Evolution of vocal learning and spoken language. Science *366*, 50–54.
33. Pfenning, A.R., Hara, E., Whitney, O., Rivas, M.V., Wang, R., Roulhac, P.L., Howard, J.T., Wirthlin, M., Lovell, P.V., Ganapathy, G., et al. (2014). Convergent transcriptional specializations in the brains of humans and song-learning birds. Science *346*, 1256846.
34. Stacho, M., Herold, C., Rook, N., Wagner, H., Axer, M., Amunts, K., and Güntürkün, O. (2020). A cortex-like canonical circuit in the avian forebrain. Science *369*, eabc5534.
35. Mol, C., Chen, A., Kager, R.W.J., and Ter Haar, S.M. (2017). Prosody in birdsong: A review and perspective. Neurosci. Biobehav. Rev. *81*, 167–180.
36. Goswami, U. (2011). A temporal sampling framework for developmental dyslexia. Trends Cogn. Sci. *15*, 3–10.
37. Jimenez-Fernandez, G., Gutierrez-Palma, N., and Defior, S. (2015). Impaired stress awareness in Spanish children with developmental dyslexia. Res. Dev. Disabil. *37*, 152–161.
38. Leong, V., Hämäläinen, J., Soltész, F., and Goswami, U. (2011). Rise time perception and detection of syllable stress in adults with developmental dyslexia. J. Mem. Lang. *64*, 59–73.
39. Fiveash, A., Bedoin, N., Gordon, R.L., and Tillmann, B. (2021). Processing rhythm in speech and music: Shared mechanisms and implications for developmental speech and language disorders. Neuropsychology *35*, 771–791.
40. Goswami, U. (2019). A neural oscillations perspective on phonological development and phonological processing in developmental dyslexia. Lang. Linguist. Compass *13*, e12328.
41. Nayak, S., Gustavson, D.E., Wang, Y., Below, J.E., Gordon, R.L., and Magne, C.L. (2022). Test of Prosody via Syllable Emphasis (“TOPsy”): Psychometric Validation of a Brief Scalable Test of Lexical Stress Perception. Front. Neurosci. *16*, 765945.
42. Hausen, M., Torppa, R., Salmela, V.R., Vainio, M., and Särkämö, T. (2013). Music and speech prosody: a common rhythm. Front. Psychol. *4*, 566.
43. Morrill, T.H., McAuley, J.D., Dilley, L.C., and Hambrick, D.Z. (2015). Individual differences in the perception of melodic contours and pitch-accent timing in speech: Support for domain-generality of pitch processing. J. Exp. Psychol. Gen. *144*, 730–736.
44. Niarchou, M., Gustavson, D.E., Sathirapongsasuti, J.F., Anglada-Tort, M., Eising, E., Bell, E., McArthur, E., Straub, P., 23andMe Research Team, McAuley, J.D., et al. (2022). Genome-wide association study of musical beat synchronization demonstrates high polygenicity. Nat. Hum. Behav. *6*, 1292–1309.
45. Ladányi, E., Persici, V., Fiveash, A., Tillmann, B., and Gordon, R.L. (2020). Is atypical rhythm a risk factor for developmental speech and language disorders? WIREs Cogn. Sci. *11*.
46. Chan, J.S., and Wade-Woolley, L. (2016). Explaining phonology and reading in adult learners: Introducing prosodic awareness and executive functions to reading ability. J. Res. Read. *41*, 42–57.
47. Gustavson, D.E., Coleman, P.L., Wang, Y., Nitin, R., Petty, L.E., Bush, C.T., Mosing, M.A., Wesseldijk, L.W., Ullén, F., 23 and Me Research Team, et al. (2023). Exploring the genetics of rhythmic perception and musical engagement in the Vanderbilt Online Musicality Study. Ann. N. Y. Acad. Sci. *1521*, 140–154.
48. Purcell, S., Neale, B., Todd-Brown, K., Thomas, L., Ferreira, M.A.R., Bender, D., Maller, J., Sklar, P., de Bakker, P.I.W., Daly, M.J., and Sham, P.C. (2007). PLINK: A Tool Set for Whole-Genome Association and Population-Based Linkage Analyses. Am. J. Hum. Genet. *81*, 559–575.
49. Conomos, M.P., Miller, M.B., and Thornton, T.A. (2015). Robust Inference of Population Structure for Ancestry Prediction and Correction of Stratification in the Presence of Relatedness. Genet. Epidemiol. *39*, 276–293.
50. Conomos, M.P., Reiner, A.P., Weir, B.S., and Thornton, T.A. (2016). Model-free Estimation of Recent Genetic Relatedness. Am. J. Hum. Genet. *98*, 127–148.
51. Loh, P.-R. (2016). Reference-based phasing using the Haplotype Reference Consortium panel. Nat. Genet. *48*, 1443–1448.
52. Taliun, D., Harris, D.N., Kessler, M.D., Carlson, J., Szpiech, Z.A., Torres, R., Taliun, S.A.G., Corvelo, A., Gogarten, S.M., Kang, H.M., et al. (2021). Sequencing of 53,831 diverse genomes from the NHLBI TOPMed Program. Nature *590*, 290–299.
53. Fuchsberger, C., Abecasis, G.R., and Hinds, D.A. (2015). minimac2: faster genotype imputation. Bioinformatics *31*, 782–784.
54. Li, H., Handsaker, B., Wysoker, A., Fennell, T., Ruan, J., Homer, N., Marth, G., Abecasis, G., Durbin, R.; and 1000 Genome Project Data Processing Subgroup (2009). The Sequence Alignment/Map format and SAMtools. Bioinformatics *25*, 2078–2079.
55. Sofer, T., Zheng, X., Gogarten, S.M., Laurie, C.A., Grinde, K., Shaffer, J.R., Shungin, D., O'Connell, J.R., Durazo-Arvizo, R.A., Raffield, L., et al. (2019). A fully adjusted two-stage procedure for rank-normalization in genetic association studies. Genet. Epidemiol. *43*, 263–275.
56. Ghoussaini, M., Mountjoy, E., Carmona, M., Peat, G., Schmidt, E.M., Hercules, A., Fumis, L., Miranda, A., Carvalho-Silva, D., Buniello, A., et al. (2021). Open Targets Genetics: systematic identification of trait-associated genes using large-scale genetics and functional genomics. Nucleic Acids Res. *49*, D1311–D1320.
57. Mountjoy, E., Schmidt, E.M., Carmona, M., Schwartzentruber, J., Peat, G., Miranda, A., Fumis, L., Hayhurst, J., Buniello, A., Karim, M.A., et al. (2021). An open approach to systematically prioritize causal variants and genes at all published human GWAS trait-associated loci. Nat. Genet. *53*, 1527–1533.
58. de Leeuw, C.A., Mooij, J.M., Heskes, T., and Posthuma, D. (2015). MAGMA: Generalized Gene-Set Analysis of GWAS Data. PLoS Comput. Biol. *11*, e1004219.
59. Bryois, J., Skene, N.G., Hansen, T.F., Kogelman, L.J.A., Watson, H.J., Liu, Z., Eating Disorders Working Group of the

Psychiatric Genomics Consortium, International Headache Genetics Consortium, 23andMe Research Team, Brueggeman, L., et al. (2020). Genetic identification of cell types underlying brain complex traits yields insights into the etiology of Parkinson's disease. Nat. Genet. *52*, 482–493.
60. Gordon, R.L., Ravignani, A., Hyland Bruno, J., Robinson, C.M., Scartozzi, A., Embalabala, R., Niarchou, M., 23andMe Research Team, Cox, N.J., and Creanza, N. (2021). Linking the genomic signatures of human beat synchronization and learned song in birds. Philos. Trans. R. Soc. Lond. B Biol. Sci. *376*, 20200329.
61. Andalman, A.S., and Fee, M.S. (2009). A basal ganglia-forebrain circuit in the songbird biases motor output to avoid vocal errors. Proc. Natl. Acad. Sci. USA *106*, 12518–12523.
62. Person, A.L., Gale, S.D., Farries, M.A., and Perkel, D.J. (2008). Organization of the songbird basal ganglia, including area X. J. Comp. Neurol. *508*, 840–866.
63. de Leeuw, C.A., Stringer, S., Dekkers, I.A., Heskes, T., and Posthuma, D. (2018). Conditional and interaction gene-set analysis reveals novel functional pathways for blood pressure. Nat. Commun. *9*, 3768.
64. Aguet, F., Anand, S., Ardlie, K.G., Gabriel, S., Getz, G.A., Graubert, A., Hadley, K., Handsaker, R.E., Huang, K.H., Kashin, S., et al. (2020). The GTEx Consortium atlas of genetic regulatory effects across human tissues. Science *369*, 1318–1330.
65. Ge, T., Chen, C.-Y., Ni, Y., Feng, Y.-C.A., and Smoller, J.W. (2019). Polygenic prediction via Bayesian regression and continuous shrinkage priors. Nat. Commun. *10*, 1776.
66. Buniello, A., MacArthur, J.A.L., Cerezo, M., Harris, L.W., Hayhurst, J., Malangone, C., McMahon, A., Morales, J., Mountjoy, E., Sollis, E., et al. (2019). The NHGRI-EBI GWAS Catalog of published genome-wide association studies, targeted arrays and summary statistics 2019. Nucleic Acids Res. *47*, D1005–D1012.
67. Holliman, A.J., Wood, C., and Sheehy, K. (2012). A cross-sectional study of prosodic sensitivity and reading difficulties. J. Res. Read. *35*, 32–48.
68. Mundy, I.R., and Carroll, J.M. (2016). Which prosodic skills are related to reading ability in adulthood? In Linguistic Rhythm and Literacy (John Benjamins Publishing), pp. 3–23. https://doi.org/10.1075/tilar.17.03mun.
69. Wood, C. (2006). Metrical stress sensitivity in young children and its relationship to phonological awareness and reading. J. Res. Read. *29*, 270–287.
70. Godel, M., Robain, F., Journal, F., Kojovic, N., Latrèche, K., Dehaene-Lambertz, G., and Schaer, M. (2023). Prosodic signatures of ASD severity and developmental delay in preschoolers. Npj Digit. Med. *6*, 99.
71. Yang, Y., Fairbairn, C., and Cohn, J.F. (2013). Detecting Depression Severity from Vocal Prosody. IEEE Trans. Affect. Comput. *4*, 142–150.
72. Patel, S., Oishi, K., Wright, A., Sutherland-Foggio, H., Saxena, S., Sheppard, S.M., and Hillis, A.E. (2018). Right Hemisphere Regions Critical for Expression of Emotion Through Prosody. Front. Neurol. *9*, 224.
73. Roberts, V.J., Ingram, S.M., Lamar, M., and Green, R.C. (1996). Prosody impairment and associated affective and behavioral disturbances in Alzheimer's disease. Neurology *47*, 1482–1488.
74. Wade-Woolley, L., Wood, C., Chan, J., and Weidman, S. (2021). Prosodic Competence as the Missing Component of Reading Processes Across Languages: Theory, Evidence and Future Research. Sci. Stud. Read. *26*, 165–181.
75. Whalley, K., and Hansen, J. (2006). The role of prosodic sensitivity in children's reading development. J. Res. Read. *29*, 288–303.
76. Magne, C., Jordan, D.K., and Gordon, R.L. (2016). Speech rhythm sensitivity and musical aptitude: ERPs and individual differences. Brain Lang. *153–154*, 13–19.
77. Resources, A. of G. TTLL1. https://www.alliancegenome.org/gene/RGD:1309124.
78. Huang, C.-C., Fornage, M., Lloyd-Jones, D.M., Wei, G.S., Boerwinkle, E., and Liu, K. (2009). Longitudinal Association of PCSK9 Sequence Variations With Low-Density Lipoprotein Cholesterol Levels: The Coronary Artery Risk Development in Young Adults Study. Circ. Cardiovasc. Genet. *2*, 354–361.
79. Jones, S.E., Lane, J.M., Wood, A.R., van Hees, V.T., Tyrrell, J., Beaumont, R.N., Jeffries, A.R., Dashti, H.S., Hillsdon, M., Ruth, K.S., et al. (2019). Genome-wide association analyses of chronotype in 697,828 individuals provides insights into circadian rhythms. Nat. Commun. *10*, 343.
80. Sakaue, S., Kanai, M., Tanigawa, Y., Karjalainen, J., Kurki, M., Koshiba, S., Narita, A., Konuma, T., Yamamoto, K., Akiyama, M., et al. (2021). A cross-population atlas of genetic associations for 220 human phenotypes. Nat. Genet. *53*, 1415–1424.
81. McCarthy, M.I. (2010). Association of Variants at UMOD with Chronic Kidney Disease and Kidney Stones—Role of Age and Comorbid Diseases. PLoS Genet. *6*.
82. Grahn, J.A. (2009). The role of the basal ganglia in beat perception: neuroimaging and neuropsychological investigations. Ann. N. Y. Acad. Sci. *1169*, 35–45.
83. Kasdan, A., Gordon, R.L., and Lense, M.D. (2022). Neurophysiological Correlates of Dynamic Beat Tracking in Individuals With Williams Syndrome. Biol. Psychiatry Cogn. Neurosci. Neuroimaging *7*, 1183–1191.
84. Merchant, H., Grahn, J., Trainor, L., Rohrmeier, M., and Fitch, W.T. (2015). Finding the beat: a neural perspective across humans and non-human primates. Philos. Trans. R. Soc. Lond. B Biol. Sci. *370*, 20140093.
85. Nozaradan, S., Schwartze, M., Obermeier, C., and Kotz, S.A. (2017). Specific contributions of basal ganglia and cerebellum to the neural tracking of rhythm. Cortex *95*, 156–168.
86. Schwartze, M., Keller, P.E., Patel, A.D., and Kotz, S.A. (2011). The impact of basal ganglia lesions on sensorimotor synchronization, spontaneous motor tempo, and the detection of tempo changes. Behav. Brain Res. *216*, 685–691.

HGGA, Volume 7

Supplemental information

Genome-wide investigation of prosody perception: Shared genetic influences between speech rhythm, musical rhythm, and reading traits

Alyssa C. Scartozzi, Youjia Wang, Peyton L. Coleman, Ximena León Du'Mottuchi, Tara L. Henechowicz, Daniel E. Gustavson, Lauren E. Petty, Heather M. Highland, Nicole Creanza, Cyrille L. Magne, Rosa S. Gísladóttir, Nancy J. Cox, Jennifer E. Below, Srishti Nayak, and Reyna L. Gordon

Supplemental Information

Supplemental Methods

Genome-wide association analysis of speech rhythm perception without the inclusion of covariates in the linear model

Since GWAS model covariates were used during our phenotype transformation step, we ran an additional GWAS without the covariates. A GWAS was performed with the transformed prosody perception scores in PLINK2[1] using a linear regression model using the flag --glm allow-no-covar. Sentinel variants were identified as the most significant variant within a +/- MB window with a minor allele count greater than 30.

Biological associations with speech rhythm perception genes in GWAS Catalog

To better characterize our genome-wide association loci, we queried our top signals (*p*-value < 5.00×10^{-6}) within the GWAS Catalog.[2] First, we filtered the GWAS Catalog (release date: 2024-03-01) to only include results that surpassed genome-wide significance, *p*-value < 5.00×10^{-8}. Next, we used our speech rhythm perception genome-wide significant and suggestive genes that were mapped using the Open Targets Genetics Variant-to-Gene (V2G) pipeline,[3,4] Table 2) in our GWAS Catalog search. Trait associations for these 13 genes were then manually categorized into 21 trait categories (Table S1a), where only 11 of our prosody-associated genes were found within the GWAS Catalog. Our speech rhythm perception findings and their associations

within the GWAS Catalog can be found in Table S1b. The number of unique speech rhythm perception genes found within each of the 20 trait categories can be found in Figure S1.

Figure S1. Unique number of speech rhythm perception-associated genes found within GWAS Catalog trait categories. Human body figure showing the number of unique speech rhythm perception-associated genes from our GWAS analysis associated with manually categorized broad trait categories in the GWAS Catalog (release date: 2024-03-01). Eleven of the suggestive signals (p-value < 5.00×10^{-6}) were found within the GWAS Catalog. Trait categories and GWAS traits can be found within Table S1.

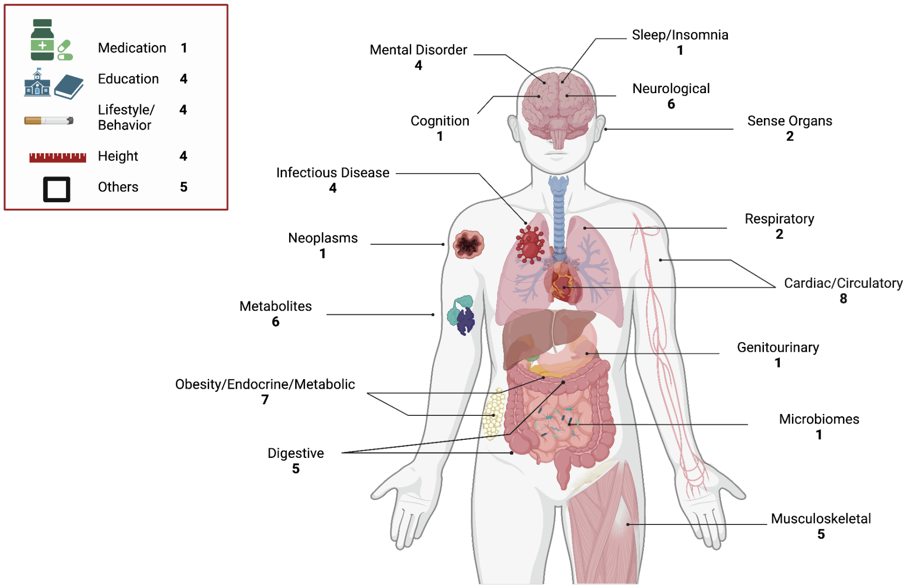

Conditional gene-set enrichment analysis of the birdsong gene sets in human speech rhythm perception

We performed the conditional gene-set enrichment analysis in MAGMA (v.1.09).[5] For our conditional gene-set enrichment analysis, average brain expression values from GTEx v8[6] was used as a gene property covariate for the birdsong gene sets with unadjusted p-values < 0.05, Singing versus Silence (Area X) and the Area X overlap.

PGS analysis in individuals of European genetic ancestry

Since our discovery GWASs were performed in individuals of European genetic ancestry, we performed PGS analyses restricting the speech rhythm perception dataset to individuals of European genetic ancestry (N = 1,501). All analyses used the European LD reference from 1000 Genomes Project Phase 3, with default auto-phi settings.

Concordance analysis with discovery GWAS and speech rhythm perception GWAS results

We performed concordance analyses to assess if there is increased directional consistency in overlapping SNPs between our speech rhythm perception GWAS and our discovery GWAS that demonstrated significant PGS, word reading[7] and beat synchronization.[8] Concordance analyses were performed in LD pruned subsets of SNPs using the following *p*-value thresholds between both sets of summary statistics: 0.05, 0.005, and 0.0005. LD pruning was performed using the plink[1] --indep-pairwise command with a window size of 10kb, step size of 1, and r2 threshold of 0.2. For a given *p*-value threshold, this analysis defines the concordance rate as the number of genetic variants with the same direction of effect divided by the total number of shared genetic variants between the two datasets. A one-sample t-test was performed to determine the concordance rate was significantly higher than expected by chance. For more information on implementation of this method see [9,10].

GWAS Power Calculations

To compute quantitative trait GWAS power, we leveraged the R package gwas-power.[11] We used the power_beta_maf function using a sample size of 1,501, beta value of 0.10 to 0.50, minor allele frequency 0.05 to 0.25, and *p*-value of 5.00 x 10^{-8}.

Supplemental Results

Genome-wide association analysis without covariates

A genome-wide association on speech rhythm perception was performed without using covariates in our linear model in 1,501 unrelated individuals of European genetic ancestry. The genomic inflation factor, λ, was 1.0117 (Figure S2). No variants surpassed genome-wide significance (*p*-value < 5.00 x 10^{-8}) and 13 loci reached suggestive significance (*p*-value < 5.00 x 10^{-6}, Table S2).

Figure S2. Manhattan plot and Q-Q plot for genome-wide association analysis of speech rhythm perception without including covariates in the linear model. Genome-wide association analysis included 1,501 individuals of European genetic ancestry and 6,778,702 variants. Genome-wide significance *p*-value 5.00 x 10^{-8} is indicated by the red line. Results showed thirteen loci of interest at a suggestive significance *p*-value threshold < 5.00 x 10^{-6} (indicated by the blue line). Q-Q plot x axis represents expected $\log_{10} p$ and the y axis represents observed $-\log_{10} p$.

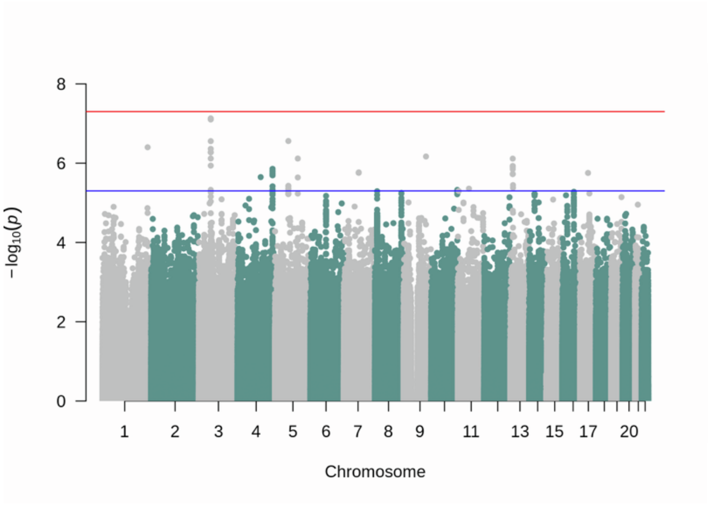

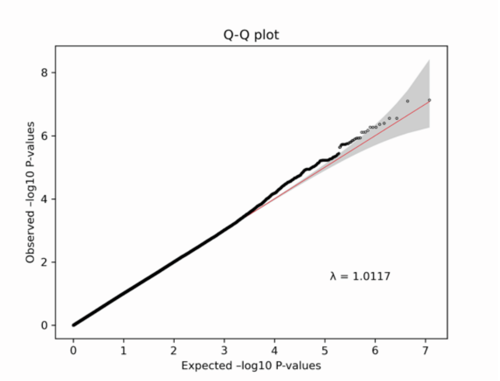

Table S2. Suggestive significant signals

associated with speech rhythm perception without including covariates in our linear model. All signals presented are identified as the most significant variant found within a +/- 1 MB window. 'Rsid': SNP, 'CHR': chromosome, 'POS_b37': position in hg37, 'BETA': the effect, 'EA': effect allele, 'NEA': non-effect allele, 'EAF': effect allele frequency, 'SE': standard error, 'Functional Gene(s)': most likely implicated functional gene that was mapped using Open Targets Genetics, '*p*-value': association *p*-value.

rsid	CHR	POS_b37	BETA	EA	NEA	EAF	SE	Functional Gene(s)	*p*-value
rs56702966	3	60958999	0.313	C	T	0.110	0.058	*FHIT*	7.41E-08
rs6886492	5	68425699	-0.238	G	A	0.188	0.046	*SLC30A5*	2.77E-07
rs80288146	1	231061747	0.610	T	C	0.024	0.120	*TTC13*	3.97E-07
rs146064482	9	114369425	-0.686	T	G	0.018	0.137	*GNG10*	6.79E-07
rs67704630	5	116506760	0.400	C	T	0.054	0.081	NA	7.64E-07
rs3000634	13	31269282	0.311	G	A	0.095	0.063	*ALOX5AP*	7.69E-07
rs6837755	4	178680187	-0.186	A	G	0.325	0.038	*AGA*	1.39E-06
rs139396308	7	76384707	-0.764	A	G	0.013	0.159	*POMZP3*	1.71E-06
rs138312553	17	41023343	0.599	G	A	0.021	0.125	*AOC3*	1.77E-06
rs146770027	4	118198184	0.856	C	A	0.010	0.180	NA	2.25E-06
rs798971	13	32758834	0.192	A	G	0.261	0.041	*NAA50P1*	3.57E-06
rs150928534	11	57464253	0.613	A	G	0.019	0.133	NA	4.39E-06
rs12774548	10	132984505	-0.260	T	G	0.117	0.057	*TCERG1L*	4.74E-06

Speech rhythm perception associated genes found within the GWAS Catalog

From the 14 suggestive significant signals within our speech rhythm perception GWAS (*p*-value < 5×10^{-6}), 13 genes were mapped via the Open Targets Genetics V2G pipeline[3,4] (Table 2). To assess which trait categories may be associated with our speech rhythm perception-associated genes, we queried the GWAS Catalog[2] (Table S1 and Figure S1). From the 13 speech rhythm perception-associated genes, only 11 were found within the GWAS Catalog. We found that eight speech rhythm perception genes were found to be previously associated with Cardiac/Circulatory traits, seven with Obesity/Endocrine/Metabolic traits, and six with Metabolic traits. We further found that

six speech rhythm perception genes were found to be previously associated with Neurological traits, and four with Mental Disorders, with three genes overlapping between these two categories. GWAS trait categorization can be found within Table S1a. The full results for the number of unique speech rhythm perception-associated genes with trait categories can be found within Figure S1 and Table S1b.

Gene-set enrichment analysis controlling for average brain expression

Results for the conditional gene-set analysis,[5] which used average brain expression as a gene property covariate, show that human speech rhythm perception's enrichments singing vs silence in Area X and the Area X overlap gene sets remain nominally significant when controlling for average brain expression levels as a gene property, *p*-value < 0.05 (Table S3). These results do not surpass Bonferroni correction for the number of gene sets tested (7 gene sets).

Table S3. Conditional gene-set analysis on the two nominally significant gene-set analysis results. This follow-up analysis controlled for average brain expression as a gene property covariate. 'Gene Set': the gene-set being tested, 'NGENES': the number of genes found within each gene-set, 'BETA': the regression coefficient, 'BETA_STD': the predicted change in Z-value given a change of one standard deviation in the predictor gene-set, 'SE': the standard error of the regression coefficient, '*p*-value': the *p*-value.

Gene Set	NGENES	BETA	BETA STD	SE	*p*-value
Set 3: Singing vs. silence (Area X)	3090	0.026	0.010	0.014	0.033
Area X Overlap	460	0.056	0.009	0.033	0.048

PGS analysis of word reading, beat synchronization, and voice pitch variability in individuals of European genetic ancestry

To directly match the genetic ancestry to our discovery GWASs of word reading, beat synchronization and voice pitch variability, we performed PGS analyses predicting the genetic predisposition of our discovery GWAS trait on speech rhythm perception scores solely in individuals of European genetic ancestry (N = 1,501). We found that the genetic predisposition of word reading[7] and beat synchronization[8] explains speech rhythm perception scores (Table S3, Figures S2a and S2b). However, we found that the genetic predisposition of voice pitch variability[12] did not explain speech rhythm perception scores (Table S3, Figures S2c). All analyses controlled for age, sex, and the first five PCs.

Table S4. Speech rhythm perception skills predicted by polygenic scores for word reading, beat synchronization, and voice pitch variability in individuals of European Genetic Ancestry (N = 1,501). PGS testing controlled for age, sex, and the first five PCs. 'Polygenic Score': the discovery GWAS used to score speech rhythm perception individuals, 'β': effect, '95% CI': 95% confidence intervals, '*p*-value': polygenic score testing *p*-value.

Polygenic Score	β	95% CI	*p*-value
Word Reading	0.09791	[0.045, 0.150]	2.44×10^{-4}
Beat Synchronization	0.144	[0.096, 0.192]	5.91×10^{-9}
Voice Pitch Variability	0.03184	[-0.017, 0.080]	1.99×10^{-1}

Figure S3. Decile plots showing polygenic scores of (a) word reading, (b) beat synchronization, and (c) voice pitch variability on the x-axis, and behavioral speech rhythm perception scores on the y-axis, in N = 1,698. Polygenic scores were derived from GWAS results reported in Eising et al. (2022), Niarchou et al., (2022), and Gisladottir et al. (2023), respectively. Polygenic scores and behavioral scores with standard error bars are visualized for N = 1,501 individuals from European genetic ancestry.

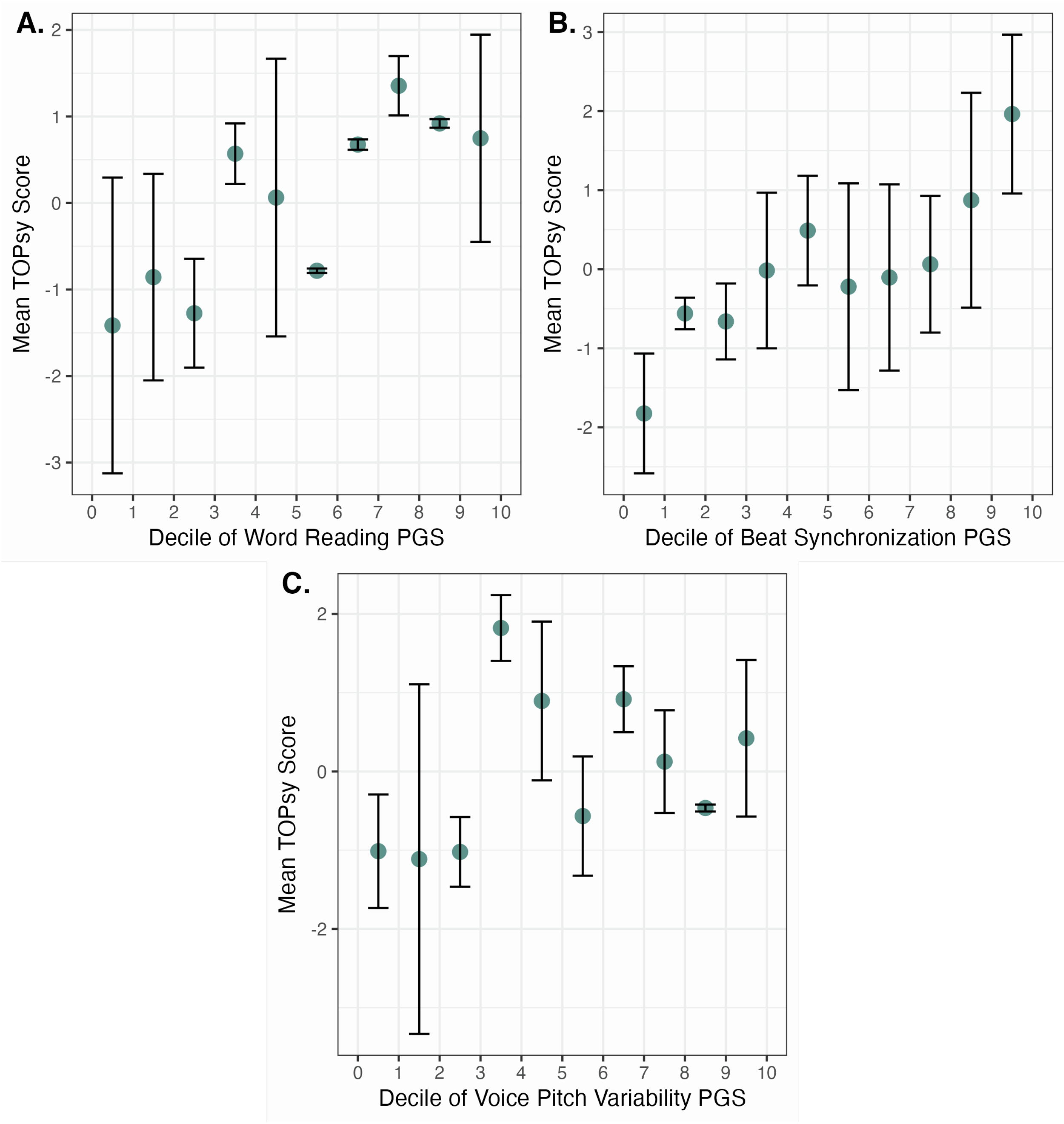

Concordance analysis results between our discovery GWAS and speech rhythm perception GWAS results

At the *p*-value threshold of 0.05 for beat synchronization and speech rhythm perception, we did observe significant concordance (concordance rate = 0.545, *p*-value = 3.857×10^{-14}, Table S5). However, our results do not demonstrate statistically

significant concordant effects at the other *p*-value thresholds for beat synchronization and speech rhythm perception. Further, we do not see statistically significant concordant effects for word reading and speech rhythm perception (Table S5).

Table S5. Concordance rates for speech rhythm perception and beat synchronization and speech rhythm perception and word reading. Concordance rate is defined as the number of overlapping LD pruned SNPs in the same direction divided by the total number of SNPs overlapping within a *p*-value threshold.

Comparison tested	*p*-value threshold	Total SNP overlap	Concordance rate	Concordance *p*-value
Speech rhythm perception and beat synchronization	0.05	6778	0.545	3.857×10^{-14}
	0.005	155	0.568	0.054
	0.0005	2	1.0	0.25
Speech rhythm perception and word reading	0.05	5154	0.505	0.222
	0.005	71	0.549	0.238
	0.0005	0	-	-

Power Calculation Results

Based on our power calculations, we are ~80% powered to detect a beta of 0.38 for variants with an MAF of 0.1, and a beta of 0.26 for variants with an MAF 0.25 (Figure S4).

Figure S4. Power calculation distribution by Minor Allele Frequency. X-axis represents Beta and y-axis represents power.

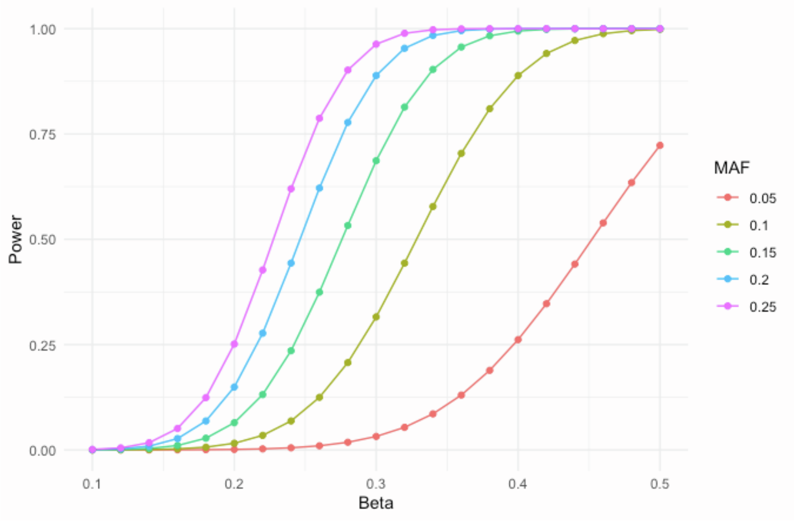

References

1. Purcell, S. *et al.* PLINK: A Tool Set for Whole-Genome Association and Population-Based Linkage Analyses. *Am. J. Hum. Genet.* **81**, 559–575 (2007).
2. Buniello, A. *et al.* The NHGRI-EBI GWAS Catalog of published genome-wide association studies, targeted arrays and summary statistics 2019. *Nucleic Acids Res.* **47**, D1005–D1012 (2019).
3. Mountjoy, E. *et al.* An open approach to systematically prioritize causal variants and genes at all published human GWAS trait-associated loci. *Nat. Genet.* **53**, 1527–1533 (2021).
4. Ghoussaini, M. *et al.* Open Targets Genetics: systematic identification of trait-associated genes using large-scale genetics and functional genomics. *Nucleic Acids Res.* **49**, D1311–D1320 (2021).
5. de Leeuw, C. A., Stringer, S., Dekkers, I. A., Heskes, T. & Posthuma, D. Conditional and interaction gene-set analysis reveals novel functional pathways for blood pressure. *Nat Commun* **9**, 3768 (2018).
6. Aguet, F. *et al.* The GTEx Consortium atlas of genetic regulatory effects across human tissues. *Science* **369**, 1318–1330 (2020).
7. Eising, E. *et al.* Genome-wide analyses of individual differences in quantitatively assessed reading- and language-related skills in up to 34,000 people. *Proc. Natl. Acad. Sci.* **119**, e2202764119 (2022).
8. Niarchou, M. *et al.* Genome-wide association study of musical beat synchronization demonstrates high polygenicity. *Nat. Hum. Behav.* **6**, 1292–1309 (2022).

9. Shaw, D. M. *et al.* Phenome risk classification enables phenotypic imputation and gene discovery in developmental stuttering. *Am. J. Hum. Genet.* **108**, 2271–2283 (2021).

10. Polikowsky, H. G. *et al.* Large-scale genome-wide analyses of stuttering. *Nat. Genet.* **57**, 1835–1847 (2025).

11. Visscher, P. M. *et al.* 10 Years of GWAS Discovery: Biology, Function, and Translation. *Am. J. Hum. Genet.* **101**, 5–22 (2017).

12. Gisladottir, R. S. *et al.* Sequence variants affecting voice pitch in humans. *Sci. Adv.* **9**, eabq2969 (2023).
